# Functional Assays Combined with Pre-mRNA-Splicing Analysis Improve Variant Classification and Diagnostics for Individuals with Neurofibromatosis Type 1 and Legius Syndrome

**DOI:** 10.1155/2023/9628049

**Published:** 2023-02-15

**Authors:** Hannie Douben, Marianne Hoogeveen-Westerveld, Mark Nellist, Jesse Louwen, Marian Kroos-de Haan, Mattijs Punt, Babeth van Ommeren, Leontine van Unen, Peter Elfferich, Esmee Kasteleijn, Yolande van Bever, Margreethe van Vliet, Rianne Oostenbrink, Jasper J. Saris, Anja Wagner, Yvette van Ierland, Tjakko van Ham, Rick van Minkelen

**Affiliations:** ^1^Department of Clinical Genetics, Erasmus University Medical Center, Rotterdam, Netherlands; ^2^Department of Pediatrics, Erasmus University Medical Center, Rotterdam, Netherlands; ^3^ENCORE Expertise Center for Neurodevelopmental Disorders, Erasmus University Medical Center, Rotterdam, Netherlands

## Abstract

Neurofibromatosis type 1 (NF1) and Legius syndrome (LS) are caused by inactivating variants in *NF1* and *SPRED1*. *NF1* encodes neurofibromin (NF), a GTPase-activating protein (GAP) for RAS that interacts with the *SPRED1* product, Sprouty-related protein with an EVH (Ena/Vasp homology) domain 1 (SPRED1). Obtaining a clinical and molecular diagnosis of NF1 or LS can be challenging due to the phenotypic diversity, the size and complexity of the *NF1* and *SPRED1* loci, and uncertainty over the effects of some *NF1* and *SPRED1* variants on pre-mRNA splicing and/or protein expression and function. To improve *NF1* and *SPRED1* variant classification and establish pathogenicity for *NF1* and *SPRED1* variants identified in individuals with NF1 or LS, we analyzed patient RNA by RT-PCR and performed *in vitro* exon trap experiments and estimated NF and SPRED1 protein expression, RAS GAP activity, and interaction. We obtained evidence to support pathogenicity according to American College of Medical Genetics guidelines for 73/114 variants tested, demonstrating the utility of functional approaches for *NF1* and *SPRED1* variant classification and NF and LS diagnostics.

## 1. Introduction

Detecting and classifying genetic variants are key to diagnosing genetic disorders. Clinical and genetic investigations, *in silico* predictions, and population data are routinely used in genome diagnostics to detect and establish pathogenicity of identified genetic variants. Nonetheless, the number of variants of unknown clinical significance (VUS) identified can equal the number of (likely) pathogenic variants, preventing a conclusive diagnosis and hindering genetic counselling [1]. Analysis of mRNA expression, pre-mRNA splicing, and protein function can help establish variant pathogenicity but are not routinely applied by diagnostic laboratories.

Neurofibromatosis type 1 (NF1; MIM# 162200) is an autosomal dominant disorder characterized by café-au-lait macules, Lisch nodules, axillary and inguinal freckling, cutaneous neurofibromas, and a wide range of patient specific symptoms [2–4]. NF1 has an incidence of ˜1/3500 and is caused by inactivation of the *NF1* tumour suppressor [5]. Due to the relatively high incidence and large size of the *NF1* gene, many VUS are identified preventing a final diagnosis and preventing optimal care of NF patients.

The canonical 12 kb *NF1* mRNA transcript, NM_000267.3, encodes a 2818 amino acid (320 kDa) GTPase-activating protein (GAP) called neurofibromin (NF) that acts on GTPases of the RAS family. Loss or inactivation of *NF1* results in increased RAS signaling and the development of lesions characteristic for NF1 [6]. Recent studies indicate that NF dimerizes in a head-to-tail orientation and that this dimerization is important for NF activation [7–10].

Legius syndrome (LS; MIM# 611431) is an autosomal dominant disorder characterized by cafe-au-lait macules, axillary and inguinal freckling, lipomas, and macrocephaly, learning disabilities, and developmental delay [11]. LS has an incidence of ˜1/75000 and is caused by inactivation of *SPRED1* that encodes the Sprouty-related protein with an EVH (Ena/Vasp homology) domain 1 (SPRED1). SPRED1 recruits the NF dimer to the plasma membrane to stimulate the GTPase activity of membrane-bound RAS [8, 12]. The functional relationship between NF and SPRED1 helps explain the phenotypic overlap between NF1 and LS. Indeed, while some amino acid substitutions impair NF RAS GAP activity to cause NF1, other changes that do not affect RAS GAP activity cause NF1 by disrupting the interaction with SPRED1 [13]. Similarly, changes to SPRED1 disrupt the interaction with NF and cause LS [14–16]. In addition to direct effects on the catalytic GAP activity and NF-SPRED1 binding, the effects of amino acid changes on the expression and stability of the NF dimer are also critical for NF function [17].

Molecular genetic analysis can establish a diagnosis of NF1 or LS: the identification of an inactivating change, such as a frameshift or nonsense variant that causes a premature stop codon and/or nonsense-mediated mRNA decay provides strong evidence to support pathogenicity. Variants that affect pre-mRNA splicing or introduce damaging changes into the NF or SPRED1 proteins are more difficult to classify. In our center, DNA-based molecular screening identified 371 *NF1* or *SPRED1* VUS (accounting for approximately 10% of all cases) ([18]; unpublished data). The American College of Medical Genetics and Genomics (ACMG) has provided guidelines for the interpretation of genetic variants [19]. Strong evidence for classifying *NF1* and *SPRED1* variants as pathogenic can be obtained by performing functional experiments (ACMG criterium PS3) [14, 16, 17, 20–23]. To provide individuals from our NF1/LS cohort with certainty regarding their affection status and follow-up and to facilitate prenatal diagnostics, we initiated functional assessment of *NF1* pre-mRNA splicing and NF-SPRED1 function and implemented these tests in our diagnostic laboratory. In addition, for cases where no candidate pathogenic variant was identified by DNA-based molecular screening, we applied RNA-sequencing to help identify variants that affect the *NF1* or *SPRED1* transcripts [24].

We tested 114 *NF1* and *SPRED1* variants. The effects of 38 variants on *NF1* pre-mRNA splicing and 76 variants on NF-SPRED1 function were investigated. In 11 cases, both pre-mRNA splicing and NF1-SPRED1 function were analyzed. The combination of RNA and protein studies enabled us to fully investigate the likely effects of the different variants. For some variants, mRNA-splicing analysis was required to identify the correct protein variant to test in the functional assays. For others, the demonstration of abnormal *NF1* pre-mRNA splicing made the testing of NF-SPRED1 protein function redundant. The results of the functional experiments, together with clinical and genetic data, were used to (re) classify the variants, following ACMG guidelines. Our integrated approach, combining testing of both pre-mRNA splicing and protein function in a routine *NF1* diagnostic testing setting, allowed (re) classification of two-thirds of the variants tested as (likely) pathogenic.

## 2. Materials and Methods

### 2.1. Editorial Policies and Ethical Considerations

Informed consent was provided by all subjects, as required by the institutional review board of the Erasmus Medical Center, and according to standard diagnostic protocols.

### 2.2. Patient Assessment and Selection of Variants for Testing

The Erasmus MC Department of Clinical Genetics NF1/LS cohort consists of >4900 index cases suspected of NF1 or LS based on the international clinical diagnostic criteria [25] for whom DNA has been submitted for genetic testing of *NF1* and/or *SPRED1*. Variants were classified using the available clinical and genetic data resulting in the identification of >2267 pathogenic or likely pathogenic *NF1* or *SPRED1* variants and >370 VUS ([18]; unpublished data). Variants were selected for functional testing following requests received from the consultant clinical geneticist.

Splice site prediction software (Alamut Visual Plus, version 1.5.1; Sophia Genetics) was used to identify variants likely to affect pre-mRNA splicing and the assay method was determined by the availability of patient RNA and/or the complexity of the predicted/observed splice abnormalities. In cases where a nonsynonymous variant was predicted to disrupt splicing, we first analyzed the putative effects on mRNA synthesis prior to deciding whether to investigate effects on protein function. For assay validation, additional variants, either from our own cohort or from literature, were selected for comparison, as detailed in Supplementary Tables S1-S3. We tested 11 variants that have been classified as pathogenic and/or subjected to functional evaluation: NM_000267.3(NF1) p.Leu90Pro [26], p.Met992del [17, 27], p.Met1149Val [17], p.Asp1217Tyr [14], p.Arg1276Gly [28], p.Lys1423Glu [17, 23, 29], p.Asp1623Gly [17], and p.Arg1809Cys [17]; NM_152594.2(SPRED1) p.Val44Asp, p.Thr102Met [14], and p.Ser105Ala [16]. In addition, we tested 3 likely benign variants from our cohort: p.Asn1229Ser, p.Pro1232Ser, and Ile1478Val. These variants were identified in individuals for whom another pathogenic, germ-line *NF1* variant, was identified (data not shown). Nomenclature for all the reported variants is according to HGVS guidelines [30].

### 2.3. Constructs, Antibodies, and Cell-Lines

*NF1* minigene exon trap constructs and NF expression plasmids were generated using standard cloning techniques [31], Gibson assembly [32], and/or site-directed mutagenesis (see Supplementary Materials for details). All constructs were verified by sequencing of the complete insert and at least 2 independent, verified clones per variant were used to prepare separate plasmid DNA stocks for the functional experiments. Nucleotide and amino acid numbering are according to reference transcripts NM_000267.3(NF1) and NM_152594.2(SPRED1), unless specified otherwise.

Antibodies were from Cell Signaling Technology (Danvers, U.S.A.) (rabbit anti-HA; mouse anti-HA; 9B11 mouse anti-myc), Invitrogen (mouse anti-V5), Sigma-Aldrich (St. Louis, U.S.A.) (mouse and rabbit anti-FLAG), and LI-COR Biosciences (Lincoln, U.S.A.) (goat anti-rabbit 680 nm and goat anti-mouse 800 nm conjugates). Anti-FLAG affinity beads were from Sigma-Aldrich; glutathione-sepharose was from GE Healthcare (Uppsala, Sweden).

HEK 293 T and COS-7 cells were maintained in Dulbecco's Modified Eagle Medium (DMEM) (Lonza, Verviers, Belgium) containing 10% fetal calf serum, 50 U/ml penicillin and 50 *μ*g/ml streptomycin in a humidified 37°C, 5-10% CO_2_ incubator.

### 2.4. Assessment of the Effects of NF1 Variants on NF1 Pre-mRNA Splicing in Patient Material

Reverse transcriptase (RT) PCR was performed on 1-2 *μ*g total RNA as described in the Supplementary Materials.

### 2.5. In Vitro Assessment of the Effects of NF1 Variants on NF1 Pre-mRNA Splicing

Exon trap experiments were performed as described previously [33, 34]. See Supplementary Materials for details.

### 2.6. In Vitro Assessment of RAS GAP Activity

To estimate RAS GAP activity, HA-H-RAS was expressed together with either wild-type (WT) or variant NF in COS-7 or HEK 293 T cells. GTP-bound RAS was subsequently isolated using glutathione-agarose beads coated with recombinant GST-RAF-RBD [35]. See Supplementary Materials for details.

### 2.7. In Vitro Assessment of the NF1-SPRED Interaction

To investigate the NF-SPRED interaction, FLAG-SPRED1 (WT or variant) and NF (WT or variant) were coexpressed in HEK 293 T cells. NF-SPRED1 complexes were immunoprecipitated using anti-FLAG affinity beads (Sigma-Aldrich). See Supplementary Materials for details.

## 3. Results

### 3.1. Assessment of the Effects of NF1 Variants on NF1 Pre-mRNA Splicing

**Figure 1:**
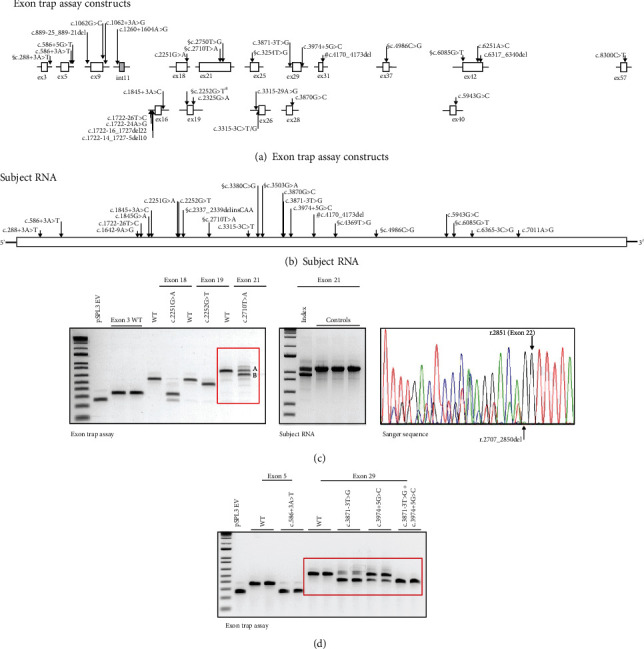
Analysis of *NF1* pre-mRNA splicing. (a) Schematic overview of *NF1* exon trap constructs derived as part of this study, showing the approximate location of the tested variants. Exonic sequences are shown as boxes and intronic sequences as horizontal lines; intronic sequence representing a pseudoexon is indicated by the shaded box. § indicates a variant also subjected to functional assessment (see Supplementary Table S2). Variant nomenclature is according to reference transcript NM_000267.3, except ¶: reference transcript NM_001042492.2. (b) Approximate location of the *NF1* variants investigated with RT-PCR and Sanger sequencing in patient RNA in relation to the NF1 reference transcript (NM_000267.3). § indicates a variant also subjected to functional assessment (see Supplementary Table S2). Note that the c.3380C>G and c.3503G>A variants were identified in *cis* in an individual with NF1. (c) Exon trap and RT-PCR analysis of the NM_000267.3(NF1): c.2710T>A variant. Agarose gel electrophoresis of the NF1 exon 21 wild-type (WT) and c.2710T>A exon trap RT-PCR products (left, outlined in red: A indicates the canonical exon 21 product; B indicates the product obtained due to the cryptic splice site at c.2706 created by the c.2710T>A substitution), RT-PCR products from RNA derived from the index patient and 3 control individuals (center), and an electropherogram of the Sanger sequence analysis of the RT-PCR products, confirming the abnormal r.2707_2850del transcript (right). (d) Exon trap and RT-PCR analysis of the NM_000267.3(NF1): c.3871-3T>G and c.3974+5G>C variants. Both variants were identified in *cis* in an individual with NF1. Agarose gel electrophoresis of the NF1 exon 29 wild-type (WT) and c.3871-3T>G, c.3974+5G>C, and c.3871-3T>G+c.3974+5G>C exon trap RT-PCR products (indicated with the red box). Note the complete loss of the wild-type product from the constructs containing both substitutions.

We investigated the effects of 38 *NF1* variants on pre-mRNA splicing (Figure 1 and Supplementary Materials, Table S1). For 30 variants, we performed *in vitro* exon trapping (Figure 1(a)). Subject RNA was available for testing of 15 of these variants, allowing confirmation of the exon trap results (Figure 1(b)). For 8 additional variants, RT-PCR and Sanger sequencing of subject RNA were carried out directly, without performing exon trap experiments (Figure 1(b)). We compared the variant exon trap constructs with the corresponding wild-type (WT) *NF1* exon (Figure 1(a)). The WT construct usually revealed a single, predominant product corresponding to the expected trapped exon (Figures 1(c) and 1(d)); although for a few exons, a minor, less intense, RT-PCR product corresponding to skipping of the WT exon was observed. The variant constructs showed either no difference, an abnormal splice product, or a combination of different products (Figures 1(c) and 1(d)).

We detected one or more abnormal *NF1* splice products, either *in vitro*, in subject RNA, or in both for 25/38 variants (66%). Exon skipping (type I defect) [36] was observed for 20 variants; but pseudoexon incorporation (type II defects), exon truncation due to utilization of a noncanonical splice site (type III defects), and intron retention (type IV defects) were also observed (Supplementary Materials, Table S1; Figure 1(c)). In 5 cases, abnormal splicing resulted in an in-frame deletion, including the nonsynonymous c.2710A>T p.(Cys904Ser) variant. To determine whether the *NF1* c.288+3 A>T, r.205_288del, p.(Arg69_Gly96del) and c.2710A>T, r.2707_2850del, p.(Cys904_Val951del) variants affected NF activity, protein function assessment was performed. In 7 cases, RNA analysis indicated that a missense change prevented canonical *NF1* pre-mRNA splicing, making assessment of NF function redundant. We did not observe an effect on *NF1* pre-mRNA splicing, either *in vitro* or in subject RNA for 13 variants. In total, analysis of *NF1* pre-mRNA splicing assisted in the classification of 25/38 variants (66%) as likely pathogenic; the remaining variants were subsequently confirmed as likely benign (3 cases; data not shown), affected protein function (5 cases; see below), or remained VUS (4 cases) (Supplementary Materials, Table S1).

### 3.2. In Vitro Assessment of the Effects of NF1 Variants on NF RAS GAP Activity

**Figure 2:**
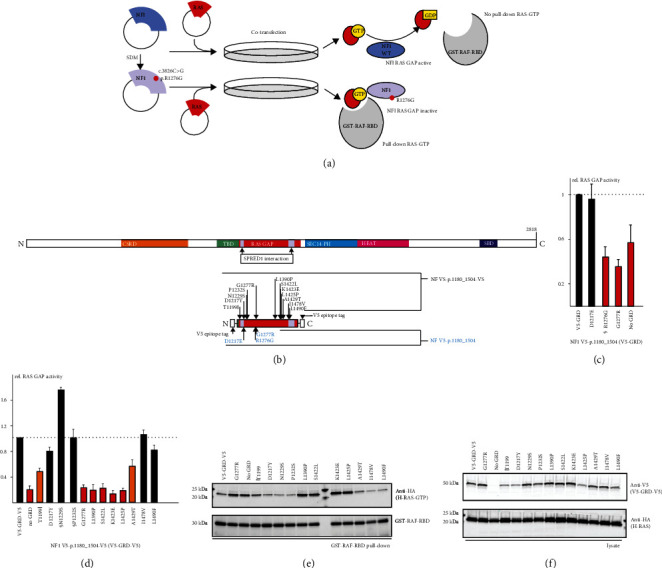
*In vitro* functional assessment of RAS GAP activity of the NF GRD. (a) Schematic overview of the pull-down assay for NF RAS GAP activity (see Materials and Methods and Supplementary Materials for details). Briefly, variants were introduced into the wild-type (WT) NF expression construct by site-directed mutagenesis (SDM), cotransfected into mammalian cells in culture together with a RAS reporter expression construct and, after 5 minute stimulation with EGF, the cells were lysed and GTP-bound RAS was subjected to GST-RAF-RBD pull-down with glutathione-agarose beads. Lysate and pull-down fractions were analyzed by immunoblotting. (b) Schematic overview of NF (top), showing the relative positions of the proposed functional domains (CSRD: orange; TBD: green; SPRED1 interaction: violet; SEC14-PH: cyan; HEAT: pink; SBD: blue) and the NF GRD expression constructs used in this study (below). Amino acid changes are given according to reference transcript NM_000267.3. Variants derived in the NF V5-p.1180_1504 expression construct [22] are indicated in cyan; other variants were derived from the WT NF p.V5-1180_1504-V5 expression construct. (c) Relative RAS GAP activity of the NF p.V5-1180_1504 variants. HA-H-RAS signals in the pull-down fractions were determined in 3 independent experiments. The mean estimated RAS GAP activity is shown relative to the WT (V5-GRD; = 1.0). Error bars represent the standard error of the mean. Variants with significantly reduced RAS GAP activity are shown as red bars (see main text for details). The RAS GAP inactive p.Arg1276Gly pathogenic variant (R1276G) [27] is indicated with §. (d) Relative RAS GAP activity of the NF p.V5-1180_1504 variants. The pull-down assay was performed as in (c) and RAS GAP activity was estimated relative to the WT (V5-GRD-V5; = 1.0) in at least 3 independent experiments. Error bars represent the standard error of the mean. Variants with no evidence for RAS GAP activity are shown as red bars; variants with reduced RAS GAP activity are shown as orange bars; active variants are shown as black bars. The likely benign p.Asn1229Ser (N1229S) and p.Pro1232Ser (P1232S) variants are indicated with §§ (see main text for details). (e) Representative immunoblot showing the GST-RAF-RBD pull-down fractions. (f) Cell lysate fractions corresponding to the samples shown in (e).

The ability of the NF GAP-related domain (GRD; amino acids 1180-1504) to inactivate RAS can be determined by measuring the amount of active, GTP-bound RAS in the presence of the NF GRD [23]. To assess the pathogenicity of 2 variants that were not predicted to affect splicing, we determined the RAS GAP activity of the NF GRD using a pull-down assay for GTP-bound RAS (Figure 2(a)). We identified the *NF1* c.3829G>C, p.(Gly1277Arg) and c.3651T>A, p.(Asp1217Glu) variants in the NF GRD in 2 individuals with NF1 and introduced both variants and the pathogenic *NF1* c.3826C>G, p.(Arg1276Gly) variant into a NF V5-p.1180_1504 expression construct [23] (Figure 2(b)). The p.Arg1276Gly and p.Gly1277Arg variants lacked RAS GAP activity, as estimated from the levels of GTP-bound RAS (RAS-GTP) in the pull-down fraction (Figure 2(c)), supporting likely pathogenicity of the *NF1* c.3829G>C, p.(Gly1277Arg) but not c.3651T>A, p.(Asp1217Glu) substitution.

Expression levels of the wild-type and variant NF V5-p.1180_1504 proteins were low (data not shown). To enhance NF GRD expression and detection, we modified the NF V5-p.1180_1504 construct by altering the sequence preceding the initiation codon to correspond to the Kozak consensus and by introducing a C-terminal V5-epitope tag. We observed robust expression of the resulting WT NF V5-p.1180_1504-V5 protein and therefore derived 12 *NF1* variants identified in our NF1 cohort, including p.(Gly1277Arg) and p.(Asp1217Glu), in the NF V5-p.1180_1504-V5 expression construct (Figure 2(b)). We determined the RAS GAP activity of the expressed NF V5-p.1180_1504-V5 proteins with the pull-down assay (Figures 2(d)–2(f)). In 5 cases, the variant lacked RAS GAP activity: levels of GTP-bound RAS (RAS-GTP) were not significantly different to those in the absence of NF V5-p.1180_1504-V5 (*P* > 0.025; Student's paired *t*-test with the Bonferroni correction; shown as red bars in Figure 2(d)). In 2 cases, RAS GAP activity was significantly reduced compared to WT NF V5-p.1180_1504-V5, but significantly increased compared to the absence of NF V5-p.1180_1504-V5 (*P* < 0.025; Student's paired *t*-test with the Bonferroni correction; orange bars in Figure 2(d)), suggesting that these variants impaired RAS GAP activity but did not inactivate the GRD completely. The remaining 5 variants retained full RAS GAP activity (*P* > 0.025; Student's paired *t*-test with the Bonferroni correction; black bars in Figure 2(d)).

### 3.3. In Vitro Assessment of the Effects of NF1 Variants on NF P.1_2069 RAS GAP Activity

**Figure 3:**
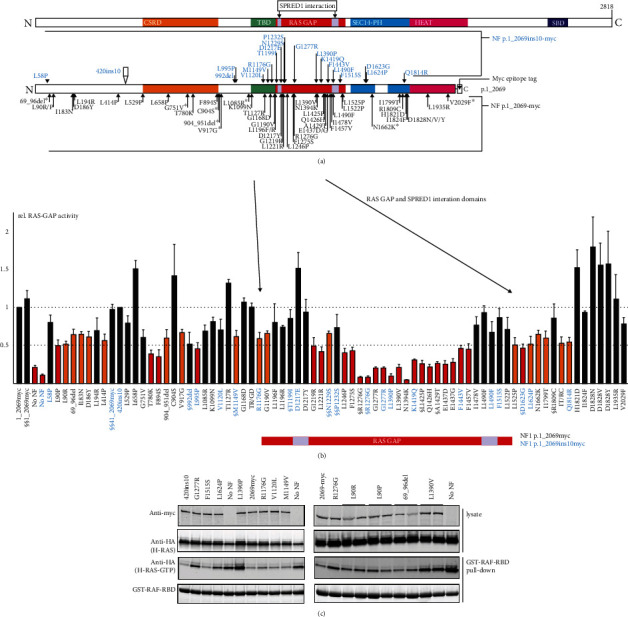
*In vitro* functional assessment of NF RAS GAP activity. (a) Schematic overview of neurofibromin (NF) (top) and the expressed truncated NF proteins used for the functional experiments (below). Amino acid changes are given according to reference transcript NM_000267.3. The C-terminal region absent from the expressed NF p.1_2069-myc and p.420ins10-myc proteins is indicated and the approximate positions of the variants are shown, including the 10 amino acid insertion NF p.420insSerThrPheLysHisGlyLeuGlyThrAla (420ins10) that differentiates the two wild-type (WT) proteins. Variants derived from the WT NF p.1_2069ins10-myc construct are cyan; variants derived from the WT NF p.1_2069-myc construct in black. To determine the RAS GAP activity of the variants, the scheme shown in Figure 2(a) was used. Briefly, NF p.1_2069-myc or NF p.1_2069ins10-myc variants were coexpressed with HA-H-RAS. RAS GAP activity relative to the corresponding WT (NF p.1_2069-myc or p.420ins10; = 1.0) was estimated in a pull-down assay using recombinant GST-RAF-RBD in at least 4 independent experiments. (b) Quantification of *NF1* variant RAS GAP activity. Variants with >50% reduction in activity (*P* < 0.05) are shown as red bars; variants with <50% reduction (*P* < 0.05) as orange bars; active variants (no evidence for reduced RAS GAP activity) (*P* > 0.05) are shown as black bars. The extent of the RAS GAP and SPRED1 interaction domains is indicated below the *x*-axis and by the thick arrows. Variants previously shown to have impaired RAS GAP activity are indicated with §; likely benign variants are indicated with §§ (see Figure 2 and main text for details). TR/GD: NF p.Thr1172Arg/Gly1168Asp, double *cis* variant; IT/RC: NF1 p.Ile1799Thr/Arg1809Cys, double *cis* variant. Variants compared to the WT NF p.2069myc protein are listed below the *x*-axis in black; variants compared to the WT NF p.420ins10 protein in cyan. Error bars represent the standard error of the mean. (c) Representative immunoblot showing the lysate (above) and GST-RAF-RBD pull-down (below) fractions.

Many *NF1* VUS identified in our cohort mapped outside the NF GRD. Attempts to introduce nucleotide changes into a full-length *NF1* expression construct were unsuccessful. However, we were able to introduce variants into 2 expression constructs encoding the N-terminal 2069 amino acids of NF (Figure 3(a)). The only difference between these 2 WT constructs was the inclusion of sequences corresponding to a neuron-specific *NF1* transcript encoding a 10 amino acid insertion (NM_000267.3(NF1) p.420insSerThrPheLysHisGlyLeuGlyThrAla; [37]. We referred to the proteins expressed from these constructs as NF p.2069myc and NF p.420ins10myc, respectively. Some initial experiments were conducted using the WT NF p.420ins10myc construct. However, most variants were derived from the WT NF p.2069myc construct as the encoded protein corresponded better with the product of the NM_000267.3 reference transcript. We did not detect significant differences in RAS GAP activity between the WT p.2069myc and p.420ins10myc proteins (Figures 3(b) and 3(c)). To further validate the assay, we investigated the correlation between NF expression levels and the estimated RAS GAP activity (Supplementary Figure S1A-C). Consistent with previous studies [17, 38], the NF signal and the estimated RAS GAP activity were dependent on the amount of NF expression construct used in the transfection experiments. Under the conditions used to compare the WT and variant NF proteins, the expression of WT NF was sufficient to increase RAS GAP activity >5-fold compared to cells not expressing any exogenous NF (Supplementary Figure S1B).

We introduced 69 *NF1* variants in the WT NF expression constructs, including 12 previously tested in the NF V5-p.1180_1504 or NF V5-p.1180_1594-V5 constructs, and determined the RAS GAP activity of the variant proteins (Figure 3 and Supplementary Materials, Table S2). Some were as active as the corresponding WT NF protein, some had severely attenuated RAS GAP activity, and others had intermediate levels of activity. This made it difficult to assign an exact cut-off value to identify pathogenic, inactivating variants. Therefore, we devised an empirical scheme to categorize the variants (Supplementary Figure S1B). We compared the mean RAS GAP activities of the variants to WT NF. If the mean RAS GAP activity was <50% of the WT (*P* < 0.05, Student's paired *t*-test), then we considered it evidence for disruption of NF RAS GAP activity and supporting evidence for pathogenicity (ACMG criteria PS3). This was the case for 22 variants (Figure 3(b); variants shown as red bars). Of these, 17 (77%) mapped to the GRD (Figure 3(b)), confirming the importance of this region for NF RAS GAP activity. With one exception, the results with the NF p.1180_1504-V5 and V5-p.1180_1504-V5 proteins were consistent. We did not detect significant impairment of RAS GAP activity by the NF p.420ins10myc p.Thr1199Ile variant, in contrast to the reduction associated with the NF V5-p.1180_1504-V5 p.Thr1199Ile variant (compare Figures 2(d) and 3(b) and see Discussion section).

In 16 cases, RAS GAP activity was significantly reduced compared to WT NF (*P* > 0.05; Student's paired *t*-test), but was >50% of the WT value (Figure 3(b); orange bars). We did not consider this sufficient evidence to support pathogenicity. The remaining variants did not show evidence for impaired RAS GAP activity: mean activity was not significantly different to WT NF (*P* > 0.05; Student's paired *t*-test; Figure 3(b), black bars).

### 3.4. In Vitro Assessment of the Effects of NF1 and SPRED1 Variants on the NF-SPRED1 Interaction

**Figure 4:**
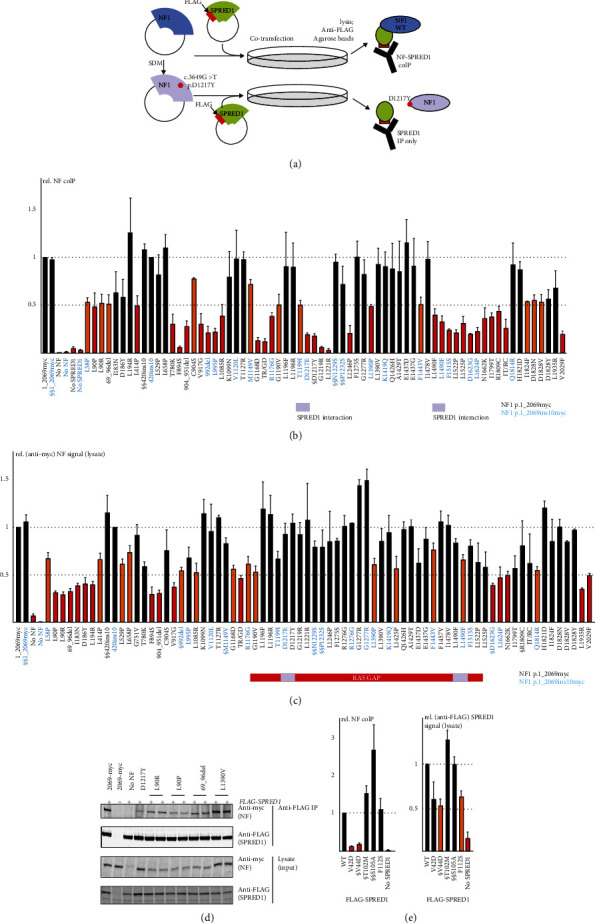
Functional assessment of the effects of *NF1* and *SPRED1* variants on NF and SPRED1 expression and on NF-SPRED1 coimmunoprecipitation (NF coIP). Amino acid changes are given according to reference transcripts NM_000267.3(*NF1*) and NM_152594.2(*SPRED1*). (a) Schematic overview of the *in vitro* functional assessment of the NF1-SPRED1 interaction by NF coIP. Variants were introduced into the wild-type (WT) expression construct by site-directed mutagenesis (SDM) and the NF p.1_2069-myc or p.1_2069ins10-myc and FLAG-SPRED1 expression constructs cotransfected into mammalian cells. NF-SPRED1 complexes were isolated using anti-FLAG agarose beads. Lysate and coIP fractions were subsequently analyzed by immunoblotting. Signals for the variants relative to the NF p.1_2069-myc (1_2069myc), NF p.1_2069ins10-myc (420ins10), or FLAG-SPRED1 WT proteins (= 1.0) were determined in at least 3 independent experiments. (b) Quantification of the NF coIP signals for the *NF1* variants. Variants with >50% reduction in signal (*P* < 0.05) are shown as red bars; variants with <50% reduction (*P* < 0.05) as orange bars; variants with comparable signals to WT NF (*P* > 0.05) are shown in black (see main text for details). The SPRED1 interaction domains [14] are indicated below the *x*-axis in mauve. The p.Asp1217Tyr (D1217Y) variant previously shown to impair NF-SPRED1 binding is indicated with §; likely benign variants are indicated with §§ (see main text for details). TR/GD: NF p.Thr1127Arg/Gly1168Asp, double *cis* variant; IT/RC: NF1 p.Ile1799Thr/Arg1809Cys, double *cis* variant. Variants compared to the WT NF p.1_2069myc protein are shown in black under the *x*-axis; variants compared to the WT NF p.420ins10 protein in cyan. Error bars represent the standard error of the mean. Note the effect of 8 variants, p.Phe1515Ser, p.Leu1522Pro, p.Leu1525Pro, p.Asp1623Gly, p.Leu1624Pro, Asn1662Lys, Ile1799Thr, and Arg1809Cys, that map distal of the SPRED1 interaction domain but clearly reduce NF coIP. (c) Quantification of the signals for the *NF1* variants in the cell lysates. Variants with >50% reduction (*P* < 0.05) in the NF coIP signal compared to the corresponding WT (= 1.0) are shown as red bars; variants with <50% reduction (*P* < 0.05) as orange bars; variants comparable to the WT (*P* > 0.05) are shown in black (see main text for details). The RAS GAP and SPRED1 interaction domains are indicated below the *x*-axis, as in Figure 3(b). Variants also analyzed by Hirata et al. are indicated with §; likely benign variants are indicated with §§ (see main text for details). TR/GD: NF p.Thr1127Arg/Gly1168Asp, double *cis* variant; IT/RC: NF1 p.Ile1799Thr/Arg1809Cys, double *cis* variant. Variants compared to the WT NF p.1_2069myc protein are shown in black; variants compared to the WT NF p.420ins10 protein in cyan. Error bars represent the standard error of the mean. (d) Representative immunoblot showing the anti-FLAG coIP (above) and lysate (below) fractions for selected *NF1* variants. (e) Quantification of the NF coIP (left) and cell lysate (right) signals for the *SPRED1* variants. Variants with >50% reduction in signal (*P* < 0.05) are shown as red bars; variants with <50% reduction (*P* < 0.05) as orange bars; variants showing comparable signals to WT SPRED1 (*P* > 0.05) are in black (see main text for details). Variants previously shown to impair NF-SPRED1 binding [14] are indicated with §; likely benign variants are indicated with §§ (see main text for details). Error bars represent the standard error of the mean.

Some pathogenic *NF1* variants disrupt the interaction between NF and SPRED1 without affecting RAS GAP activity [13]. We used an anti-FLAG affinity matrix to coimmunoprecipitate (coIP) the WT NF p.2069myc and p.420ins10myc proteins together with coexpressed WT FLAG-SPRED1 (Figure 4(a)) and determined whether 67 *NF1* (Figures 4(b) and 4(d) and Supplementary Table S2) and 5 *SPRED1* variants (Figure 4(e), left; Supplementary Table S3) affected NF-SPRED1 coIP. We compared the WT and variant signals in the IP fractions (Figures 4(b) and 4(e), left) and categorized the variants using the same criteria as for the RAS GAP assay: a significant reduction (*P* < 0.05, Student's paired *t*-test) of >50% in the mean NF signal in the IP fraction (NF coIP) was evidence for an effect on the NF-SPRED1 interaction, supporting pathogenicity (Supplementary Figure S1C). We did not detect significant differences in expression or NF-SPRED1 coIP between the WT p.2069myc and p.420ins10myc proteins (Figures 4(b) and 4(c)). To validate the NF coIP assay, we determined the effect of NF expression levels on the NF signals in the IP fraction (Supplementary Materials, Figure S1D-F). The NF coIP signal correlated with the NF signal in the cell lysate and was dependent on the amount of transfected NF expression construct.

NF coIP was reduced >50% for 29 *NF1* and 2 *SPRED1* variants, including the *NF1* p.Asp1217Tyr and *SPRED1* p.Val44Asp variants previously shown to disrupt the NF-SPRED1 interaction [14] (Figures 4(b) and 4(e), red bars). There was a significant reduction in the NF coIP signal for an additional 11 *NF1* variants, but the mean value was >50% of WT NF (Figure 4(b), orange bars) and we did not consider this sufficient evidence to support pathogenicity. The remaining variants did not differ from WT NF (*P* > 0.05, Student's paired *t*-test; Figures 4(b) and 4(e), left, black bars). We did not test 5 *NF1* variants that had clearly disrupted either RAS GAP activity or *NF1* pre-mRNA splicing in earlier assays (Supplementary Table S2).

### 3.5. In Vitro Assessment of the Effects of NF1 and SPRED1 Variants on the Expression and Stability of NF and SPRED1

**Figure 5:**
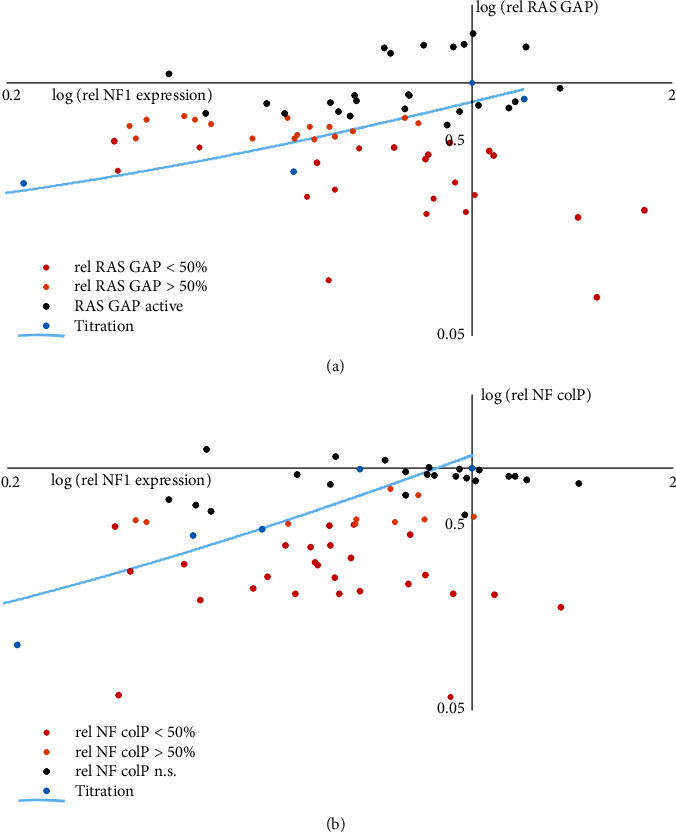
Comparison of NF variant expression, RAS GAP activity and NF coIP. (a) Scatter plot to compare *NF1* variant expression and RAS GAP activity. NF wild-type (WT) signals and the corresponding relative RAS GAP activity were determined by titrating the amount of transfected NF expression construct (see Supplementary Figure S2). WT NF expression and activity under standard assay conditions were defined as 1 (origin). Data points and the estimated trendline are shown (cyan), compared to the relative RAS GAP activity and expression of the different *NF1* variants (detailed in Figures 3(b) and 4(c), respectively, and in Supplementary Table S2). Variants with >50% reduction in activity (*P* < 0.05) are shown in red; variants with <50% reduction (*P* < 0.05) in orange; active variants (no evidence for reduced RAS GAP activity) (*P* > 0.05) are in black. (b) Scatter plot to compare NF1 variant expression and NF coIP. NF WT signals in the cell lysates and the corresponding signals in the anti-FLAG IP fractions were determined by titrating the amount of transfected NF expression construct (see Supplementary Figure S2). WT NF expression and coIP under standard assay conditions were defined as 1 (origin). Data points and the estimated trendline are shown (cyan), compared to the NF coIP and expression of the different *NF1* variants (see Figures 4(b) and 4(c) and Supplementary Table S2). Variants with >50% reduction in NF coIP (*P* < 0.05) are shown in red; variants with <50% reduction (*P* < 0.05) in orange; variants comparable to WT NF (*P* > 0.05) in black. n.s.: not significant.

Differences in RAS GAP activity and NF coIP could reflect differences in the expression and/or stability of the variant proteins. To determine the effects of *NF1* and *SPRED1* variants on NF-SPRED1 expression and stability, we compared WT and variant NF and SPRED1 signals in the cell lysates by immunoblotting (Figures 3(c) and 4(c)–4(e), right). In addition, we determined the effect of WT NF levels on both RAS GAP activity and NF coIP (Supplementary Figure S1). We compared the resulting titration curves to the expression, RAS GAP activity, and NF coIP of the *NF1* variants, as estimated under standard assay conditions (Figure 5). Notably, some variants were expressed at levels equal to or above WT, yet were still unable to inactivate RAS or were not immunoprecipitated efficiently with SPRED1.

Mean NF expression was reduced by >50% for 15 *NF1* variants (Figure 4(c), red bars). Of these, either RAS GAP activity, NF coIP, or both was reduced by >50% in 9 cases. Both RAS GAP activity and NF coIP were significantly reduced in 2 additional cases, but by <50% (see Discussion); in 2 cases, RAS GAP activity was reduced but by <50%, and in 2 cases, neither RAS GAP activity or NF coIP was significantly different to WT NF (compare Figure 4(c) with Figures 3(b) and 4(b) and see Supplementary Table S2).

## 4. Discussion

Although the diagnostic yield for NF1 and LS is >90%, the high incidence of NF1 means that many patients still lack a molecular diagnosis because either no candidate pathogenic variant or a VUS in *NF1/SPRED1* is found. To complement the *NF1* and *SPRED1* DNA test results from our laboratory, we implemented functional assays to assess 114 *NF1/SPRED1* variants in a diagnostic setting. We employed 4 functional assays: (i) analysis of subject mRNA by RT-PCR; (ii) *in vitro* exon trap analysis of *NF1* pre-mRNA splicing; (iii) *in vitro* analysis of NF RAS GAP activity; and (iv) *in vitro* analysis of NF-SPRED1 expression and interaction. We considered the evidence sufficient for reclassification of 73/114 (64%) variants as (likely) pathogenic (class 4 and 5) (Supplementary Materials, Tables S1, S2, and S3), demonstrating the utility of functional approaches for *NF1* and *SPRED1* variant classification and NF1 and LS diagnostics. The results of our experiments have been submitted to the *NF1* and *SPRED1* Leiden Open Variation Databases (https://databases.lovd.nl/shared/genes/NF1;https://databases.lovd.nl/shared/genes/SPRED1).

In contrast to laboratories that specialize in *NF1* variant detection and classification using patient RNA [21, 39], our diagnostic laboratory performs molecular screening primarily on DNA samples because direct analysis of RNA was not considered practical for routine screening in our setting [18]. The *in vitro* exon trap experiments provided a useful screen for identifying *NF1* variants likely to affect splicing, without having to resample patients. We did not observe major discrepancies between the exon trap and RT-PCR results that would have led to a different classification for any of the variants tested, consistent with other work from our laboratory [24, 40]. Furthermore, the exon trap analysis meant that the observed *in vitro* effects of a potentially pathogenic variant could be communicated prior to taking a tissue sample for confirmation. The exon trap approach also assisted in resolving allele-specific patterns of pre-mRNA splicing when phasing was not possible due to a lack of informative exonic variants. For example, expression of the WT allele sometimes prevented us from determining whether the canonical *NF1* transcript was also expressed from the variant allele. The exon trap experiments indicated whether a variant was likely to completely prevent canonical splicing or only have a partial effect. In 4 cases, there were minor differences between the *in vitro* and *in vivo* RNA data (Supplementary Materials, Table S1). However, we did not identify cases where a variant had a major effect on splicing *in vitro* but not *in vivo* or *vice versa*. Analysis of pre-mRNA splicing was also a useful screen for the functional assessments as it was not always obvious whether a variant was likely to affect splicing and/or protein function. In some cases, RNA analysis revealed abnormal *NF1* splicing, making functional assessment redundant, whereas in other cases, RNA analysis indicated that functional assessment of an in-frame deletion was indicated to establish pathogenicity. In 25/38 cases (66%), functional assessment of *NF1* pre-mRNA splicing provided sufficient evidence for us to classify the variant as (likely) pathogenic (class 4 and 5; ACMG criteria PS3).

Compared to the exon trapping and RT-PCR experiments, assessment of NF-SPRED1 function was labour-intensive, time-consuming, and had other limitations (see below), meaning that the findings had to be interpreted with caution and in the light of clinical and genetic evidence. Nonetheless, we obtained functional evidence to support pathogenicity for 46 *NF1* and 2 *SPRED1* variants, including the known pathogenic variants *NF1* p.(Leu90Pro), p.(Met992del), p.(Asp1217Tyr), p.(Arg1276Gly), p.(Lys1423Glu), p.(Asp1623Gly) and p.(Arg1809Cys), and *SPRED1* p.(Val44Asp) (Supplementary Materials, Tables S2 and S3). Our data showing loss of RAS GAP activity for the *NF1* p.Lys1423Glu variant and disruption of the NF-SPRED1 interaction for the *NF1* p.Asp1217Tyr and *SPRED1* p.Val44Asp variants were consistent with previous reports [14, 17, 23].

In contrast to a recent study that analyzed full-length murine *Nf1* variants [17], our expressed NF proteins lacked a segment of the C-terminal HEAT-repeat region that is involved in NF dimerization [7–10]. Nonetheless, robust, reproducible effects on RAS GAP activity, NF coIP, and/or NF expression/stability were observed, even though the variants were expressed at nonphysiological levels (Figures 3 and 4 and Supplementary Materials, Tables S2 and S3). Some differences in the estimated activity or expression might reflect variation in transfection efficiency, cell numbers, immunoblotting artefacts, or other processing errors, and it is possible that some variants that disrupted NF-SPRED1 function in our *in vitro* assays might retain sufficient activity *in vivo* to prevent NF1 or LS. With these caveats in mind, we devised an empirical scheme to categorize the variants. We considered a >50% reduction in either RAS GAP activity or NF coIP as functional evidence to support pathogenicity (Supplementary Figure S1). We did not consider a >50% reduction in expression/stability as sufficient evidence for pathogenicity unless it was concordant with significant disruption of RAS GAP activity and/or NF coIP (*P* < 0.05, Student's paired *t*-test) (Supplementary Materials, Tables S2 and S3). Variants that did not significantly reduce RAS GAP activity or NF coIP remained VUS, unless other genetic evidence was obtained to support or exclude pathogenicity, such as *de novo* occurrence of the variant in a sporadic case of NF1 or identification of another pathogenic variant in the same individual.

None of the variants for which we obtained evidence to support pathogenicity were identified more than once in the gnomAD (v2.1) database (https://gnomad.broadinstitute.org/) (accessed7/3/2022), and none were classified as benign or likely benign in ClinVar (https://www.ncbi.nlm.nih.gov/clinvar/) (accessed 7/3/2022). The variants were all identified in at least one individual suspected of NF1 or LS in our cohort. The remaining variants did not show sufficient evidence for an effect on NF or SPRED1 function to support pathogenicity, even though several are described as likely pathogenic in ClinVar (https://www.ncbi.nlm.nih.gov/clinvar/; Supplementary Materials, Table S2). In one case, we observed a discrepancy between the results of the RAS GAP assay with the NF V5-p.1180_1504-V5 GRD and NF p.420ins10myc protein. The NF1 p.Thr1199Ile variant impaired RAS GAP activity of the GRD but did not significantly affect RAS GAP activity of the NF p.420ins10 protein (compare Figures 2(d) and 3(b)). It is possible that the NF GRD and p.420ins10myc proteins have distinct sensitivities to changes in secondary structure. The extra scaffolding around the active site of the GRD provided by the p.420ins10myc protein might restrict structural changes and thereby help maintain RAS GAP activity. For this reason, and because the larger NF p.2069myc and p.420ins10myc proteins could also be used to not only investigate the effects of more variants but also interrogate the NF-SPRED1 interaction, we currently derive NF p.2069myc variants for functional assessment.

We observed good correlation between the variants affecting either RAS GAP activity or NF coIP and their location within or adjacent to the NF GRD and SPRED1 interaction domains (Figures 3(a) and 3(b) and 4(b)). Interestingly, we identified 8 *NF1* variants just distal of the second, C-terminal SPRED1 interaction domain that clearly disrupted NF coIP (Figure 4(b); amino acids 1522-1809). It is possible that this region of NF is important for maintaining the correct spatial orientation of the SPRED1 interaction domains. Intriguingly, we also identified a cluster of *NF1* variants, p.His1821Asp, Asp1828Asn, Asp1828Val, and Asp1828Tyr, with increased RAS GAP activity (~150%) compared to WT NF (Figure 3(b) and Supplementary Table S2). More experiments are required to investigate the significance of this finding.

In contrast to an earlier study [13], we identified variants that disrupted both RAS GAP activity and the NF-SPRED1 interaction (Supplementary Table S2; compare Figures 3(b) and 4(b)). Notably, several of these variants, *NF1* p.Thr780Lys, p.Leu995Pro, p.Gly1219Arg, p.Leu1221Arg, and p.Leu1246Pro, did not reduce NF expression (Figure 4(c) and Supplementary Table S2), suggesting that they affect NF function without destabilising the protein.

Variants classified as likely benign according to ACMG criteria did not show sufficient evidence to support pathogenicity in our functional assays (Supplementary Table S2). However, we did observe differences for variants previously classified as (likely) pathogenic [17]. According to our criteria, the p. Met992del, p.Met1149Val, and p.Arg1809Cys variants did not reduce NF RAS GAP activity sufficiently to support pathogenicity. Nonetheless, NF coIP was clearly reduced for the p.Met992del and p.Arg1809Cys variants, supporting pathogenicity (the NF-SPRED1 interaction was not assessed in [17]). The absence of the C-terminal region of NF (amino acids 2070-2818) from our expressed NF variants as well as differences between murine and human NF could account for the differences between the two studies, but we note that all 3 variants have been associated with a distinct NF1 phenotype [27, 29, 41], and it is possible that other variants with (partial) retention of NF RAS GAP activity might be associated with less severe disease. Advances in the structural and functional biology of NF will help inform decisions regarding the pathogenicity of these and other variants. Furthermore, functional analysis of a larger number of known pathogenic *NF1* and *SPRED1* variants could help establish more accurate criteria for determining likely pathogenicity and identify correlations between specific deficits in NF function and the clinical phenotype.

We did not use the results of the functional assessment to exclude pathogenicity. We only interrogated 3 aspects of NF-SPRED1 function: RAS GAP activity, the NF-SPRED1 interaction, and expression/stability. We did not investigate other putative functions of NF or SPRED1, such as phospholipid binding [42, 43], cell invasiveness [44], or the regulation of estrogen receptor dependent transcriptional activity [45]. Furthermore, we were unable to investigate *NF1* variants distal to residue 2069. Efforts to efficiently derive *NF1* variants in a full-length *NF1* expression construct are on-going in our laboratory.

Despite the limitations detailed above, our work enabled a molecular diagnosis to be made for individuals suspected of NF1 and LS in whom a VUS in *NF1* or *SPRED1* had been identified. We obtained evidence to support pathogenicity for 73/114 variants (64%) (Supplementary Materials, Tables S1, S2, and S3) and together with consideration of the clinical, population, *in silico*, and segregation data, functional testing helped establish likely variant pathogenicity in these cases. Implementation of functional testing in our laboratory has improved molecular diagnostics for individuals with NF1 and LS (Supplementary Figure S1) and facilitated appropriate monitoring, treatment, and prenatal diagnostic options for family planning. Our approach shows that the integration of assays for *NF1/SPRED1* pre-mRNA splicing and protein function allows reclassification of a significant proportion of *NF1* and *SPRED1* VUS, drastically improving molecular diagnostics for individuals and families with NF1 and LS. Furthermore, our study demonstrates that diagnostic laboratories proficient in protein-based analyses such as immunoblotting and immunoprecipitation can apply similar functional approaches for variant classification, not only for NF1 and LS but also for other specific genetic conditions.

## Data Availability

The data and materials are available from the corresponding authors upon reasonable request.

## Supplementary Materials

Supplementary Materials A file containing supplementary material pertinent to this manuscript is available. This supplementary information consists of 3 tables, 2 figures, and detailed Materials and Methods, as follows: Supplementary Table S1: summary of the assessment of effects of *NF1* variants on *NF1* pre-mRNA splicing. Supplementary Table S2: summary of the functional assessment of *NF1* variants. Supplementary Table S3: summary of the functional assessment of *SPRED1* variants. Supplementary Figure S1: NF RAS GAP activity and NF-SPRED1 coimmunoprecipitation (NF coIP) functional assay validation. Supplementary Figure S2: flow diagrams to illustrate the NF1/LS molecular diagnostic screening process in our laboratory.
