# Supplementary material for: Functional Assays Combined with Pre-mRNA-Splicing Analysis Improve Variant Classification and Diagnostics for Individuals with Neurofibromatosis Type 1 and Legius Syndrome
Source: Hum Mutat. 2023 Feb 15;2023:9628049. doi: 10.1155/2023/9628049 (PMC11918873; doi:10.1155/2023/9628049)
Supplement: Supplementary Materials — A file containing supplementary material pertinent to this manuscript is available. This supplementary information consists of 3 tables, 2 figures, and detailed Materials and Methods, as follows: Supplementary Table S1: summary of the assessment of effects of NF1 variants on NF1 pre-mRNA splicing. Supplementary Table S2: summary of the functional assessment of NF1 variants. Supplementary Table S3: summary of the functional assessment of SPRED1 variants. Supplementary Figure S1: NF RAS GAP activity and NF-SPRED1 coimmunoprecipitation (NF coIP) functional assay validation. Supplementary Figure S2: flow diagrams to illustrate the NF1/LS molecular diagnostic screening process in our laboratory. [file 9628049.f1.docx]

**Functional assays combined with pre-mRNA splicing analysis improve variant classification and diagnostics for individuals with Neurofibromatosis type 1 and Legius syndrome.**

Hannie Douben^1*^, Marianne Hoogeveen-Westerveld^1*^, Mark Nellist^1^, Jesse Louwen^1^, Marian Kroos-de Haan^1^, Mattijs Punt^1^, Babeth van Ommeren^1^, Leontine van Unen^1^, Peter Elfferich^1^, Esmee Kasteleijn^1^, Yolande van Bever^1^, Margreethe van Vliet^1^, Rianne Oostenbrink^2,3^, Jasper J. Saris^1^, Anja Wagner^1^, Yvette van Ierland^1,3^, Tjakko van Ham^1,#^, Rick van Minkelen^1,3,#^

^1.^ Department of Clinical Genetics, Erasmus University Medical Center, Rotterdam, The Netherlands

^2.^ Department of Pediatrics, Erasmus University Medical Center, Rotterdam, The Netherlands

^3.^ ENCORE Expertise Center for Neurodevelopmental Disorders, Erasmus University Medical Center, Rotterdam, The Netherlands

* these authors contributed equally

^#^ these authors contributed equally

Correspondence and requests for materials should be addressed to: Dr. T. van Ham (t.vanham@erasmusmc.nl) or Dr. R. van Minkelen (r.vanminkelen@erasmusmc.nl), Department of Clinical Genetics, Erasmus University Medical Center, Wytemaweg 80, 3015CN Rotterdam, The Netherlands.

**Supplementary Information**

The Supplementary Information consists of 3 tables, 2 figures and detailed Materials and Methods.

**Supplementary Table S1. Summary of the assessment of effects of *NF1* variants on *NF1* pre-mRNA splicing.** Overview of the *in vitro* exon trap assay results and comparison with the results of *in silico* splice site prediction and RT-PCR analysis of patient RNA (see main text and Supplementary Information for details). Nucleotide and protein numbering is according to the *NF1* reference transcript NM_000267.3, unless specified otherwise. Splicing defects are categorised according to [36]; variants for which analysis of pre-mRNA splicing did not support variant pathogenicity are shaded grey. *In silico* splice prediction was carried out in the Alamut Visual Plus software package (Sophia Genetics, version 1.5.1); variants tested for validation purposes are indicated with 'validation'. VUS: variant of uncertain clinical significance.

| **NF1 variant [NM_000267.3]** | ***in silico* splice prediction** | ***in vitro* splice assay** | **RT-PCR analysis of patient RNA** | **Type of splice defect** | **Variant classification (ACMG criteria; [19])** |
| --- | --- | --- | --- | --- | --- |
| c.288+3A>T (exon 3) | disrupts 5' donor | r.205_288del p.(Arg69_Gly96del) | r.205_288del p.(Arg69_Gly96del) | I | pathogenic (PS3, PS1, PM2_sup, PP4, PP1)(see Table S2) |
| c.586+3A>T (exon 5) | disrupts 5'donor | r.480_586del p.(Leu161Asnfs*4) | r.480_586del p.(Leu161Asnfs*4) | I | likely pathogenic (PS3, PM2_supp, PP4) |
| c.889-25_889-21del (exon 9) | unlikely to affect splicing | no effect | not done | no defect | benign (BS3, BS1, BP4) |
| c.1062G>C p.(Lys354Asn) (exon 9) | disrupts 5' donor | r.889_1062del p.(Lys297_Lys354del) | not done | I | pathogenic (PS1, PS3, PM2_sup, PP3, PP4) |
| c.1062+3A>G (exon 9) | disrupts 5' donor | r.889_1062del p.(Lys297_Lys354del) | not done | I | likely pathogenic (PS3, PM2_sup, PP1, PP3, PP4) |
| c.1260+1604A>G (intron 11) | creates cryptic 3' acceptor | r.1260ins1260+1605_1260+1646 p.(Asn420_421insLeuThrThr*) | not done | II | likely pathogenic (PS3, PM2_sup, PP3, PP4, PP5) |
| c.1642-9A>G (exon 15) | destroys canonical 3’acceptor and creates cryptic 3' acceptor | not done | r.1642-1ins1642-8_1642-1 p.(Glu547fs*10) (major product); r.1642_1721del p.(Ala548Leufs*13) (minor product) | III | likely pathogenic (PS3, PM2_sup, PP3, PP4) |
| c.1722-26T>G (exon 16) | disrupts branch point | r.1722_1845del p.(Ser575Argfs*15) | transcript switching/intron retention: NM_001128147.3 | I/IV | likely pathogenic (PS3, PM2_sup, PP4)[25] |
| c.1722-14_1722-5del10 (exon 16) | disrupts 3' acceptor | r.1722_1845del p.(Ser575Argfs*15) | not done | I | likely pathogenic (PS3, PM2_sup, PP4)[25] |
| c.1722-16_1727del22 (exon 16) | destroys 3' acceptor (validation) | r.1722_1845del p.(Ser575Argfs*15) | not done | I | pathogenic (PVS1, PS3, PM2_sup, PP3, PP4)[25] |
| c.1722-24A>G (exon 16) | disrupts branch point | r.1722_1845del p.(Ser575Argfs*15) | not done | I | likely pathogenic (PS3, PM2_sup, PP4)[25] |
| c.1845G>A p.(=) (exon 16) | disrupts 5' donor | not done | r.1722_1845del p.(Ser575Argfs*15) | I | likely pathogenic (PS3, PM2_sup, PP3, PP4) |
| c.1845+3A>C (exon 16) | disrupts 5' donor | r.1722_1845del p.(Ser575Argfs*15) | r.1722_1845del p.(Ser575Argfs*15) | I | likely pathogenic (PS3, PM2_sup, PP4) |
| c.2251G>A p.(Gly751Arg) (exon 18) | disrupts 5' donor | r.2002_2251del p.(Asp668Glufs*9) | r.2002_2251del p.(Asp668Glufs*9) | I | likely pathogenic (PS3, PM2_sup, PP3, PP4) |
| c.2252G>T p.(Gly751Val) (exon 19) | disrupts 3' acceptor | r.2252_2325del p.(Arg752Leufs*17) | r.2252_2325del p.(Arg752Leufs*17) | I | likely pathogenic (PS3, PM2_sup, PP3, PP4)(see Table S2) |
| c.2325G>A p.(=) (exon 19) | disrupts 5' donor | r.2252_2325del p.(Arg752Leufs*17) | not done | I | likely pathogenic (PS3, PM2_sup, PP3, PP4) |
| c.2337_2339delTACinsCAA p.(Thr780Lys) (exon 20) | unlikely to affect splicing | not done | no effect | no defect | likely pathogenic (PS2, PS3,PM2_sup, PP4) (see Table S2) |
| c.2710T>A p.(Cys904Ser) (exon 21) | activates cryptic 5' donor | r.2707_2850del p.(Cys904_Val951del) | r.2707_2850del p.(Cys904_Val951del) | III | likely pathogenic (PS3, PM2_sup, PP3, PP4) (see Table S2) |
| c.2750T>G p.(Val917Gly) (exon 21) | creates cryptic 5' donor | r.2750_2850del p.(Val917Glyfs*5) | not done | III | likely pathogenic (PS3, PM2_sup, PP3, PP4) (see Table S2) |
| c.3254T>G p.(Leu1085Arg) (exon 25) | unlikely to affect splicing | no effect | not done | no defect | likely pathogenic (PS3, PM2_sup, PP4) (see Table S2) |
| c.3315-29A>G | unlikely to affect splicing | no effect | not done | no defect | likely benign (BS3, BP4) |
| c.3315-3C>T (exon 26) | unlikely to affect splicing | no effect | no effect | no defect | likely benign (BS3, BP4) |
| c.3315-3C>G | disrupts 3’acceptor | r.3315_3496del p.(Tyr1106Leufs*28) | not done | I | likely pathogenic (PS3, PM2_sup, PP3, PP5) |
| c.3380C>G p.(Thr1127Arg) + c.3503G>A p.(Gly1168Asp) (in *cis*) | unlikely to affect splicing | not done | no effect | no defect | likely pathogenic (PM1, PM2_sup, PP3/BP4)(see Table S2) |
| c.3870G>C p.(Lys1290Asn) (exon 28) | destroys 5' donor | r.3870_3871ins3870+1_3870+38 p.(Lys1290Asnfs*32); r.3845_3870del p.(Lys1283Ilefs*22) | r.3870_3871ins3870+1_3870+145 p.(Lys1290Asnfs*18) (minor product); r.3870_3871ins3870+1_3870+38 p.(Lys1290Asnfs*3) (minor product); r.3845_3870del p.(Lys1283Ilefs*22) (major product) | IV | likely pathogenic (PS3, PM2_sup, PP3, PP4) |
| c.3871-3T>G (exon 29) | disrupts 3' acceptor | r. 3871_3974del p. (Tyr1292Argfs*7) (10% exon retention) | not done | I | likely pathogenic (PS3, PM2_sup, PP3, PP4) |
| c.3974+5G>C (exon 29) | destroys 5' donor | r. 3871_3974del p. (Tyr1292Argfs*7) (50% exon retention) | not done | I | likely pathogenic (PS3, PM2_sup, PP3, PP4) |
| c.3871-3T>G + c.3974+5G>C (exon 29 in *cis*) | destroy 3’ acceptor and 5’donor sites | r. 3871_3974del p. (Tyr1292Argfs*7) (no exon retention; 100% exon skipping) | not done | I | likely pathogenic (PS3, PM2_sup, PP3, PP4) |
| [NM_001042492.2] c.4170_4173del p.(Ser1391Trpfs*14)(exon 31) | creates cryptic 5' donor | r.4170_4173del p.(Ser1391Trpfs*14) | r.4170_4173del p.(Ser1391Trpfs*14) | II | likely pathogenic (PS3, PM2_sup, PP3, PP4) |
| c.4369T>G p.(Phe1457Val)(exon 34) | disrupts 3' acceptor | not done | not effect | no defect | likely pathogenic (PS3, PM2_sup, PP1, PP4) (see Table S2) |
| c.4986C>G p.(Asn1662Lys) | creates cryptic 5' donor | r.4982_5065del, p.(Cys1661_Asp1689delinsTyr) (minor product ~10%) | no effect | I | likely pathogenic (PS3, PM2_sup, PP1, PP4) (see Table S2) |
| c.5943G>C p.(Gln1981His) (exon 40) | destroys 5' donor | r.5943G>C_5944ins5943+1_[5943+70?], p.? (major product); r.5940_5943del, p.(Gln1981Leufs*9) (minor product) | r.5940_5943del, p.(Gln1981Leufs*9) | III/IV | likely pathogenic (PS3, PM2_sup, PP3, PP4) |
| c.6085G>T p.(Val2029Phe) (exon 42) | disrupts 3' acceptor | r.6085_6364del p.(Val2029Lysfs*7) | r.6085_6364del p.(Val2029Lysfs*7) | I | likely pathogenic (PS3, PM2_sup, PP1, PP4) (see Table S2) |
| c.6251A>C p.(His2084Pro) (exon 42) | activates cryptic 3' acceptor | no effect | not done | no defect | VUS (PM2_sup, PP1, PP3, PP4) |
| c.6317_6340del p.(Ile2106_Cys2113del) (exon 42) | unlikely to affect splicing | no effect | not done | no defect | VUS (PM2_sup, PM4, PP4) |
| c.6365-3C>G (exon 43) | disrupts canonical 3' acceptor and creates non-canonical 3' acceptor | not done | r.6364_6365ins6365-1_6365-2, p.(Glu2123Lysfs*7) (major product) + r.6365_6479del, p.(Glu2122Valfs*19) | III/I | likely pathogenic (PS3, PM2, PP5) |
| c.7011A>G p.(=) (exon 48) | creates cryptic 5' donor | not done | no effect | no defect | VUS (PM2, PP4) |
| c.8300C>T p.(Ser2767Phe) (exon 57) | unlikely to affect splicing | no effect | not done | no defect | VUS (PM2, PP4) |

**Supplementary Table S2. Summary of the functional assessment of *NF1* variants.** The tested variants and the estimated NF RAS GAP activity, NF-SPRED1 interaction and NF expression are shown. Data are for the NF p.2069myc expression construct, unless indicated otherwise: § NF p.420ins10; ¶ NF V5-p.1180_1504-V5. Significant reductions (*P* < 0.05 Student's paired t-test) of > 50% in RAS GAP activity, SPRED1 binding or expression are shown in red text. Significant reductions of < 50 % are indicated in orange text. Variants for which there was insufficient evidence to support pathogenicity are shaded grey. Nucleotide and protein numbering is according to *NF1* reference transcript NM_000267.3. The population frequency of the variant in gnomaAD v2.1 (when reported), along with the number of reports in Clinvar are indicated for each variant (both databases accessed 7/3/2022). Variants tested for validation purposes are indicated with 'validation'. VUS: variant of uncertain clinical significance.

| **NF1 variant**  **[NM_000267.3]** | **Classification prior to testing** | **rel RAS GAP activity (*P* value)** | **rel NF coIP (*P* value)** | **rel NF1 expression (*P* value)** | **Frequency in gnomAD v2.1; Clinvar reference** | **Classification (ACMG criteria; [19])** |
| --- | --- | --- | --- | --- | --- | --- |
| c.173T>C p.(Leu58Pro) § | VUS | 0.805 (0.113) | 0.532 (0.002) | 0.670 (0.014) | RCV000804180.3 | likely pathogenic (PM2_sup, PM5, PP1, PP3, PP4) |
| c.269T>C p.(Leu90Pro) | pathogenic [26], validation | 0.498 (0.0025) | 0.486 (0.017) | 0.290 (2.82 x 10^-6^) | RCV001062713.2 | pathogenic (PS1, PS3, PP4, PP5) |
| c.269T>G p.(Leu90Arg) | VUS | 0.517 (3.46 x 10^-5^) | 0.523 (0.004) | 0.312 (1.79 x 10^-7^) | RCV000659961.1 | pathogenic (PS3, PM2_sup, PM5, PP3, PP4, PP5) |
| c.205_288del p.(Arg69_Gly96del) | VUS | 0.645 (0.003) | 0.511 (0.004) | 0.324 (7.14 x 10^-6^) |  | pathogenic (PS1, PS3, PP1, PP4) |
| c.548T>A p.(Ile183Asn) | VUS | 0.648 (0.008) | 0.633 (0.167) | 0.384 (0.028) |  | VUS (PM2, PP3, PP4) |
| c.556G>T p.(Asp186Tyr) | VUS | 0.610 (0.003) | 0.586 (0.093) | 0.405 (0.0009) | RCV001214491.2, RCV000756436.2 | VUS (PM2, PM5, PP3, PP4) |
| c.581T>G p.(Leu194Arg) | VUS | 0.696 (0.129) | 1.262 (0.513) | 0.398 (0.003) | 1/250388; RCV000059209.1 | likely pathogenic (PM2_sup, PM5, PP3, PP4, PP5) |
| c.1241T>C p.(Leu414Pro) | VUS | 0.563 (0.002) | 0.496 (0.015) | 0.662 (0.043) |  | likely pathogenic (PS3, PM2_sup, PP3, PP4) |
| c.1260ins[1260+1617_c.1260+1646], p.(Ser420insSerThrPheLysHisGlyLeuGlyThrAla) | benign [37], validation | 1.129 (0.174) | 1.154 (0.434) | 1.248 (0.131) |  | benign |
| c.1586T>C p.(Leu529Pro) | VUS | 0.791 (0.098) | 0.818 (0.447) | 0.612 (0.006) |  | VUS (PM2_sup, PM5, PP3, PP4) |
| c.1973T>C p.(Leu658Pro) | VUS | 1.511 (0.011) | 1.101 (0.471) | 0.738 (0.009) |  | VUS (PM2_sup, PP4, BP4) |
| c.2252G>T p.(Gly751Val) | VUS | 0.607 (0.058) | not tested | 0.918 (0.553) |  | likely pathogenic (PS3, PM2_sup, PP3, PP4 (see Table 1) |
| c.2337_2339delinsCAA p.(Thr780Lys) | VUS | 0.387 (1.94 x 10^-5^) | 0.303 (0.049) | 0.585 (0.238) |  | pathogenic (PS2, PS3, PM2_sup, PP4) |
| c.2681T>C p.(Phe894Ser) | VUS | 0.354 (0.005) | 0.060 (0.0002) | 0.294 (0.012) | 1/251142; RCV001050822.2 | likely pathogenic (PS3, PM2_sup, PP3, PP4, PP5) |
| c.2707_2850del p.(Cys904_Val951del) | VUS | 0.596 (0.034) | 0.279 (1.13 x10^-8^) | 0.306 (1.50 x 10^-7^) |  | likely pathogenic (PS3, PM2_sup, PP3, PP4) |
| c.2710T>A p.(Cys904Ser) | VUS | 1.414 (0.415) | 0.777 (0.003) | 0.754 (0.382) |  | likely pathogenic (see Table 1) (PS3, PM2_sup, PP3, PP4) |
| c.2750T>G p.(Val917Gly) | VUS | 0.670 (0.004) | 0.305 (0.008) | 0.369 (0.0008) |  | likely pathogenic (PS3, PM2_sup, PP3, PP4) |
| c.2970_2972del p.(Met992del) § | pathogenic [17, 29], validation | 0.515 (0.053) | 0.212 (0.0004) | 0.542 (0.0011) | 1/250346; Clinvar 5x | pathogenic (PS3, PM2_sup, PM4, PP4, PP5) |
| c.2984T>C p.(Leu995Pro) § | VUS | 0.457 (0.003) | 0.219 (6.2 x 10^-6^) | 0.677 (0.104) | Clinvar 3x | likely pathogenic (PS3, PM2_sup, PP3, PP4) |
| c.3254T>G p.(Leu1085Arg) | VUS | 0.691 (0.057) | 0.387 (0.037) | 0.523 (0.040) | RCV000622984.1 | likely pathogenic (PS3, PM2_sup, PP4) |
| c.3297A>C p.(Lys1099Asn) | VUS | 0.814 (0.808) | 0.793 (0.525) | 1.144 (0.431) |  | VUS (PM1, PM2_sup, PP3) |
| c.3358G>C p.(Val1120Leu) § | VUS | 0.706 (0.108) | 0.987 (0.968) | 0.957 (0.890) | 6/139936; RCV001237795.2, RCV000572045.1 | likely benign (BS3, BP4) |
| c.3380C>G p.(Thr1127Arg) | VUS | 1.069 (0.418) | 0.979 (0.810) | 1.103 (0.396) |  | VUS (PM1, PM2_sup, BP4) |
| c.3445A>G p.(Met1149Val) § | pathogenic [17], validation | 0.616 (0.018) | 0.719 (0.012) | 0.830 (0.067) | 1/251376; RCV001591409.3, RCV000632403.5 | pathogenic (PS2, PS3, PP4) |
| c.3503G>A p.(Gly1168Asp) | VUS | 1.317 (0.240) | 0.130 (0.0001) | 0.558 (0.010) |  | likely pathogenic (PS3, PM1, PM2_sup, PP3) |
| c.3380C>G p.(Thr1127Arg) + c.3503G>A p.(Gly1168Asp) | VUS | 1.005 (0.742) | 0.120 (6.4 x 10^-5^) | 0.463 (0.003) |  | likely pathogenic (PS3, PM1, PM2_sup, PP3) |
| c.3526A>G p.(Arg1176Gly) § | VUS | 0.591 (0.007) | 0.386 (0.004) | 0.611 (0.043) | RCV001212119.2 | likely pathogenic (PS3, PM2_sup, PP3, PP4) |
| c.3569G>T p.(Gly1190Val) | VUS | 0.657 (0.0001) | 0.504 (0.012) | 0.528 (0.001) |  | VUS (PM2, PP4) |
| c.3586C>T p.(Leu1196Phe) | VUS | 0.801 (0.499) | 0.908 (0.840) | 1.161 (0.618) | RCV001339121.1, RCV001751663.1 | VUS (PM2_sup, PM5, PP4, PP5) |
| c.3587T>G p.(Leu1196Arg) | VUS | 0.743 (0.053) | 0.902 (0.715) | 1.136 (0.545) | RCV000568890.2, RCV000059188.1 | VUS (PM2_sup, PM5, PP4, PP5) |
| c.3596C>T p.(Thr1199Ile) § | VUS | 0.856 (0.281) | 0.504 (5.16 x 10^-5^) | 0.667 (0.006) | RCV001557292.2, RCV000703473.3 | VUS (PS3, PM2_sup, PP4, PP5) |
| c.3649G>T p.(Asp1217Tyr) | pathogenic [14], validation | 0.938 (0.719) | 0.178 (1.4 x 10^-14^) | 1.357 (0.118) |  | pathogenic (PS2, PS3, PM2_sup, PP3, PP4) |
| c.3651T>A p.(Asp1217Glu)  § | VUS | 1.515 (0.131) | 0.211 (1.13 x 10^-12^) | 0.937 (0.235) |  | pathogenic (PS2, PS3, PM2_sup, PP4) |
| c.3655G>C p.(Gly1219Arg) | VUS | 0.492 (0.020) | 0.059 (9.86 x 10^-5^) | 0.926 (0.616) | RCV001290862.1 | pathogenic (PS1, PS3, PM2_sup, PP3, PP4) |
| c.3662T>G p.(Leu1221Arg) | VUS | 0.420 (0.002) | 0.208 (0.026) | 1.080 (0.937) | RCV001046150.2 | likely pathogenic (PS3, PM2_sup, PP3, PP4, PP5) |
| c.3686A>G p.(Asn1229Ser) § | VUS, validation | 0.656 (0.007) | 0.956 (0.632) | 0.793 (0.096) | 9/251370; Clinvar 3x | likely benign (BS3, BP5, BP6) |
| c.3694C>T p.(Pro1232Ser) § | VUS, validation | 0.736 (0.223) | 0.719 (0.235) | 0.793 (0.370) | 1/251382; Clinvar 3x | likely benign (PM2_sup, BS3, BP5) |
| c.3737T>C p.(Leu1246Pro) | VUS | 0.405 (0.002) | 0.267 (0.163) | 0.851 (0.373) |  | likely pathogenic (PS3, PM2_sup, PP3, PP4) |
| c.3824T>C p.(Phe1275Ser) | VUS | 0.427 (6.95 x 10^-5^) | 1.009 (0.964) | 0.860 (0.189) |  | likely pathogenic (PS3, PM2_sup, PP3, PP4) |
| c.3826C>G p.(Arg1276Gly) | pathogenic [28], validation | 0.079  (1.36 x 10^-46^) | not tested | 1.540 (0.078) | 1/251202; Clinvar 3x | pathogenic (PS2, PS3, PM2_sup, PM5, PP3, PP4) |
| c.3829G>C p.(Gly1277Arg) | VUS | 0.202  (8.78 x 10^-24^) | 0.826 (0.378) | 1.446 (1.043 x 10^-7^) |  | likely pathogenic (PS3, PM2_sup, PM5, PP3, PP4) |
| c.4169T>C p.(Leu1390Pro) § | VUS | 0.097 (4.89 x 10^-5^) | 0.489 (0.0009) | 0.610 (0.030) | RCV001228388.2 | likely pathogenic (PS3, PM2_sup, PM5, PP3, PP4) |
| c.4168C>G p.(Leu1390Val) | VUS | 0.212 (4.83 x 10^-6^) | 0.930 (0.693) | 0.855 (0.324) | RCV000660051.3 | likely pathogenic (PS3, PM2_sup, PM5, PP3, PP4) |
| c.4182T>G p.(Asn1394Lys) | VUS | 0.044 (2.01 x 10^-8^) | not tested | 1.392 (0.033) | RCV001211430.2 | pathogenic (PS1, PS3, PM2_sup, PP4, PP5) |
| c.4255A>C p.(Lys1419Gln) § | VUS | 0.308 (0.0001) | 0.907 (0.588) | 0.945 (0.787) | RCV000059195.1 | likely pathogenic (PS3, PM2_sup, PP1, PP3, PP4, PP5) |
| c.4265C>T p.(Ser1422Leu) ¶ | VUS | 0.220 (6.21 x 10^-5^) | not tested | 1.816 (0.082) | RCV001540502.2, RCV000632429.3 | likely pathogenic (PS3, PM2_sup, PP1, PP3, PP4, PP5) |
| c.4267A>G p.(Lys1423Glu) ¶ | pathogenic [17, 23], validation | 0.134 (3.45 x 10^-7^) | not tested | 2.529 (0.022) | 3/140200; Clinvar 5 x | pathogenic (PS2, PS3, PM2_sup, PM5, PP3, PP4) |
| c.4274T>C p.(Leu1425Pro) | VUS | 0.259 (5.7 x 10^-5^) | not tested | 0.565 (0.017) | RCV001379175.1, RCV000059199.2 | likely pathogenic (PS3, PM2_sup, PP1, PP3, PP4, PP5) |
| c.4278G>C p.(Gln1426His) | VUS | 0.217 (7.55 x 10^-8^) | 0.881 (0.702) | 0.980 (0.478) | RCV000700494.2, RCV000220491.2 | likely pathogenic (PS3, PM2_sup, PM5, PP3, PP4, PP5) |
| c.4285G>A p.(Ala1429Thr) | VUS | 0.265 (2.42 x 10^-7^) | 0.857 (0.820) | 1.010 (0.469) |  | likely pathogenic (PS3, PM2_sup, PP3, PP4, PP5) |
| c.4310A>G p.(Glu1437Gly) | VUS | 0.281 (6.65 x 10^-7^) | 1.161 (0.625) | 0.623 (0.409) | 1/140226; RCV000231680.4, RCV001762518.1 | likely pathogenic (PS3, PM2_sup, PM5, PP3, PP4, PP5) |
| c.4311A>T p.(Glu1437Asp) | VUS | 0.253 (3.93 x 10^-7^) | 0.917 (0.800) | 0.876 (0.895) |  | likely pathogenic (PS3, PM2_sup, PM5, PP3, PP4) |
| c.4327T>G p.(Phe1443Val) § | VUS | 0.462 (0.001) | 0.511 (0.008) | 0.764 (0.035) |  | likely pathogenic (PS3, PM2_sup, PP4) |
| c.4369T>G p.(Phe1457Val) | VUS | 0.447 (0.001) | not tested | 1.062 (0.945) | RCV000570335.1 | likely pathogenic (PS3, PM2_sup, PP1, PP4) |
| c.4432A>G p.(Ile1478Val) | VUS, validation | 0.767 (0.092) | 0.983 (0.906) | 1.024 (0.347) | 2/251294; RCV000232755.2, RCV000164937.1 | likely benign (BS3, BP5) |
| c.4468C>T p.(Leu1490Phe) § | VUS | 0.675 (0.152) | 0.328 (0.0004) | 0.656 (0.028) | RCV001066994.2 | likely pathogenic (PS3, PM2_sup, PM5, PP4, PP5) |
| c.4544T>C p.(Phe1515Ser) | VUS | 0.865 (0.319) | 0.240 (0.0003) | 0.802 (0.091) |  | likely pathogenic (PS3, PM2_sup, PP3, PP4) |
| c.4565T>C p.(Leu1522Pro) | VUS | 0.710 (0.138) | 0.211 (0.001) | 0.631 (0.212) |  | likely pathogenic (PS3, PM2_sup, PP4) |
| c.4574T>C p.(Leu1525Pro) | VUS | 0.509 (0.013) | 0.311 (0.010) | 0.580 (0.125) | RCV001039214.2, RCV000216997.1 | likely pathogenic (PS3, PM2_sup, PP4) |
| c.4868A>G p.(Asp1623Gly) § | pathogenic [17], validation | 0.465 (0.0002) | 0.195 (1.72 x 10^-6^) | 0.390 (0.002) | RCV001298487.1 | pathogenic (PS3, PM2_sup, PM5, PP4) |
| c.4871T>C p.(Leu1624Pro) § | VUS | 0.516 (0.009) | 0.226 (0.0005) | 0.468 (0.045) | RCV001246838.2 | likely pathogenic (PS3, PM2_sup, PP4) |
| c.4986C>G p.(Asn1662Lys) | VUS | 0.648 (0.321) | 0.361 (0.029) | 0.491 (0.007) |  | likely pathogenic (PS3, PM1, PM2_sup, PP3) |
| c.5396T>C p.(Ile1799Thr) | VUS | 0.593 (0.021) | 0.377 (0.005) | 0.571 (0.085) | RCV000802831.1, RCV001024029.2 | pathogenic (PS1, PS3, PM2_sup, PP4) |
| c.5425C>T p.(Arg1809Cys) | pathogenic [17, 42], validation | 0.862 (0.505) | 0.439 (0.008) | 0.806 (0.541) | 1/140180; Clinvar 7x | pathogenic (PS1, PS3, PM2_sup, PP4) |
| c. 5396T>C p.(Ile1799Thr)  + c.5425C>T p.(Arg1809Cys) | VUS | 0.528 (0.010) | 0.258 (0.017) | 0.622 (0.347) |  | pathogenic (PS1, PS3, PM2, PP4) |
| c.5441A>G p.(Gln1814Arg) § | VUS | 0.541 (0.0007) | 0.925 (0.774) | 0.546 (0.007) | RCV000221556.1 | VUS (PM2_sup, PP4) |
| c.5461C>G p.(His1821Asp) | VUS | 1.525 (0.108) | 0.871 (0.229) | 1.206 (0.067) | RCV001024143.1 | VUS (PM2_sup, PP4) |
| c.5470A>T p.(Ile1824Phe) | VUS | 0.936 (0.163) | 0.538  (0.004) | 0.849  (0.632) |  | VUS (PM1, PM2_supp, BP4) |
| c.5482G>A p.(Asp1828Asn) | VUS | 1.794 (0.137) | 0.551 (0.00059) | 1.005 (0.078) |  | VUS (PM2_sup) |
| c.5483A>T p.(Asp1828Val) | VUS | 1.555 (0.308) | 0.533 (0.029) | 0.846 (0.614) | RCV001776007.1, RCV000797116.1 | VUS (PM2_sup, PP5) |
| c.5482G>T p.(Asp1828Tyr) | VUS | 1.571 (0.410) | 0.564 (0.051) | 0.973 (0.305) |  | VUS (PM2_sup) |
| c.5804T>G p.(Leu1935Arg) | VUS | 1.107 (0.757) | 0.682 (0.175) | 0.350 (1.10 x 10^-5^) | RCV001209336.2, RCV001024557.1 | VUS (PM2_sup,PP5) |
| c.6085G>T p.(Val2029Phe) | VUS | 0.780 (0.080) | 0.260 (0.002) | 0.492 (0.002) | 1/139240; RCV001224793.2, RCV001550330.2 | likely pathogenic (PS3, PM2_sup, PP4, PP1) |

**Supplementary Table S3. Summary of the functional assessment of *SPRED1* variants.** The tested variants and the estimated relative expression and immunoprecipitation (IP) of SPRED1 and co-immunoprecipitation (coIP) of NF p.2069myc are shown. Significant (P <0.05 Student's paired t-test) reductions of > 50% in SPRED1 IP, NF co-IP or SPRED1 expression are shown in red; significant reductions of < 50 % are indicated in orange. Variants for which there was insufficient evidence to support pathogenicity are shaded grey. Nucleotide and protein numbering is according to the *SPRED1* reference transcript NM_152594.2. The population frequency of the variant in gnomaAD v2.1 (if reported), along with the number of reports in Clinvar are indicated for each variant (both databases accessed 7/3/2022). Variants included for validation purposes are indicated with 'validation'. VUS: variant of uncertain clinical significance.

| **SPRED1 variant**  **[NM_152594.2]** | **Classification prior to testing** | **rel NF coIP (*P* value)** | **rel SPRED1 IP (*P* value)** | **rel SPRED1 expression (*P* value)** | **Frequency in gnomAD v2.1; Clinvar reference** | **Classification (ACMG criteria; [19])** |
| --- | --- | --- | --- | --- | --- | --- |
| c.125T>A p.(Val42Asp) | VUS | 0.126 (0.0004) | 0.603 (0.025) | 0.600 (0.288) |  | likely pathogenic (PS3, PM2_sup, PP4) |
| c.131T>A p.(Val44Asp) | pathogenic [14], validation | 0.184 (0.0006) | 0.463 (0.0005) | 0.528 (0.024) | RCV000001889.3 | pathogenic (PS1, PS3, PM2_sup, PP4) |
| c.305C>T p.(Thr102Met) | pathogenic [14], validation | 1.513 (0.112) | 1.407 (0.201) | 1.274 (0.224) |  | likely pathogenic (PS1, PS3, PM2_sup, PP4) |
| c.313_314delinsGC p.(Ser105Ala) | VUS [16], validation | 2.662 (0.066) | 1.532 (0.282) | 0.995 (0.949) |  | VUS |
| c.335T>C p.(Phe112Ser) | VUS | 1.095 (0.749) | 0.797 (0.306) | 0.630 (0.013) |  | VUS (PM2_sup, PP4) |

**Supplementary Figures**

**Supplementary Figure S1.** **NF RAS GAP activity and NF-SPRED1 coimmunoprecipitation (NF coIP) functional assay validation.** To estimate the effects of NF expression levels on RAS GAP activity (A - C) and NF coIP (D - F), titration experiments were performed in duplicate with varying amounts of transfected WT NF p.2069-myc expression construct.

(A) Representaive immunoblot for the NF RAS GAP assay, showing the expressed NF proteins (top), and RAS-GTP levels as determined by GST-RAF-RBD pull-down (bottom) (see Supplementary Materials and Methods for details). The amount of transfected WT NF expression construct (ng DNA) is indicated.

(B) Quantification of the RAS GAP activity. Error bars represent the standard error of the mean.

(C) Quantification of the NF signals in the cell lysates used for estimating NF RAS GAP activity. Error bars represent the standard error of the mean.

(D) Representaive immunoblot for the NF coIP assay, showing the NF and SPRED1 signals in the anti-FLAG IP (top) and cell lysate (bottom) fractions (see Supplementary Materials and Methods for details). The amount of transfected WT NF expression construct (µg DNA) is indicated.

(E) Quantification of the NF signals in the NF coIP fractions. Error bars represent the standard error of the mean.

(F) Quantification of the NF signals in the cell lysates used NF coIP. Error bars represent the standard error of the mean.


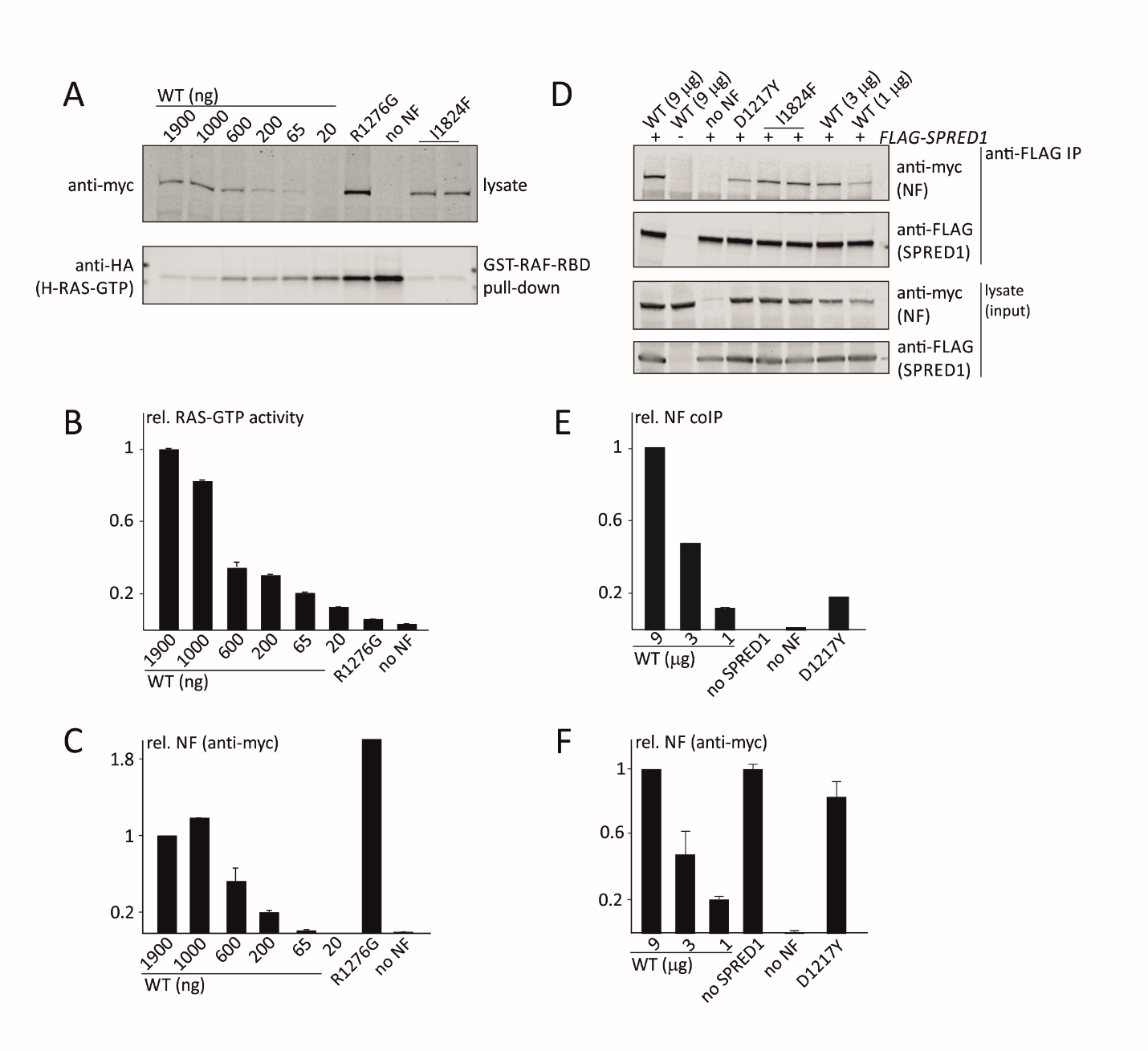


**Supplementary Figure S2.** **Flow diagrams to illustrate the NF1/LS molecular diagnostic screening process in our laboratory.**

(A) DNA samples isolated from individuals suspected of NF1/LS are submitted to exon-based molecular screening. In 2267/4900 cases (46%) a pathogenic (P) or likely pathogenic (LP) variant was identified; in an additional 371 cases (8%) variants of uncertain significance (VUS) were identified. Analysis of *NF1* pre-mRNA splicing and/or NF-SPRED1 protein function assisted in re-classification of 73/114 variants tested (64%).

(B) Empirical scheme for the categorization of *NF1* variants according to their effect on NF RAS GAP activity.

(C) Empirical scheme for the categorization of *NF1* and *SPRED1* variants according to their effect on NF-SPRED1 coimmunoprecipitation (NF coIP).

(D) Empirical scheme for the categorization of *NF1* variants according to their effect on NF expression/stability.


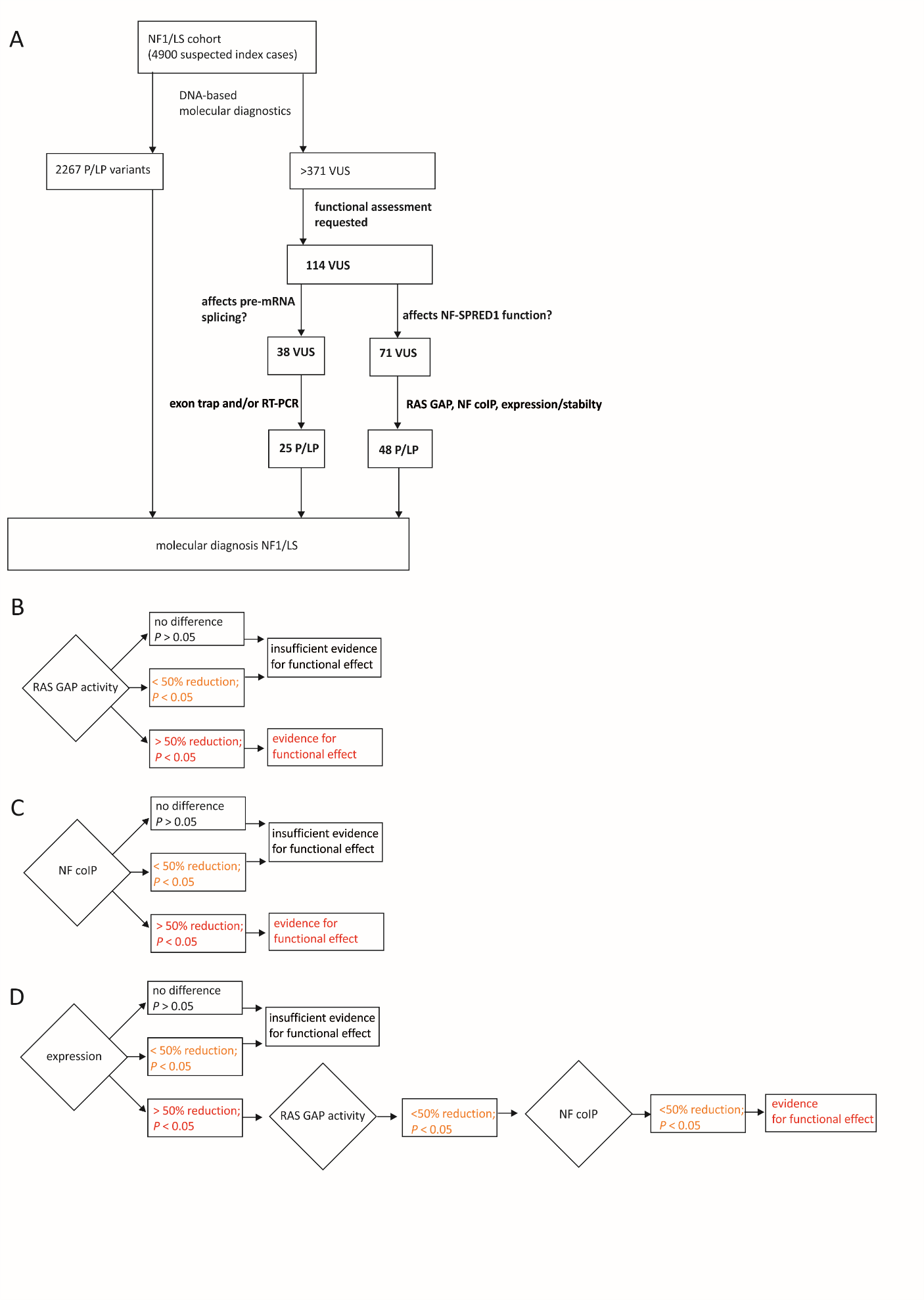


**Supplementary Materials and Methods**

*Patient assessment and selection of variants for testing*

Clinical and genetic information was extracted from the Erasmus MC Department of Clinical Genetics NF1 patient database. Nomenclature for all reported variants was according to the current HGVS guidelines [30]. Variants were classified according to ACMG guidelines [19] using the available clinical and genetic data. All individuals provided consent for testing.

*Constructs, antibodies, and cell-lines*

*NF1* minigene constructs were generated using standard cloning techniques [31], Gibson assembly [32] and/or site-directed mutagenesis (SDM). Briefly, *NF1* exons and the surrounding intronic sequences were amplified from the DNA of an individual carrying the variant of interest by PCR. The PCR products were subsequently cloned into the pSPL3 splicing vector [34] to yield constructs for both the wild-type (WT) and variant alleles. Primer sequences are available on request. In some cases variants were introduced by SDM, using the QuikChange II XL SDM kit (Agilent, Santa Clara, USA). All constructs were verified by sequencing of the entire insert.

The recombinant NF expression plasmids used for this study are shown in Figures 2 and 3. The NF V5-p.1180 _1504 expression construct, encoding the NF GRD (c.3538_4512, p.Met1180_Asn1504) and an N-terminal V5 epitope tag, was kindly provided by Dr. Thomas and Prof. Upadhyaya (Institute of Medical Genetics, Cardiff, UK)[23]. The NF V5-p.1180_1504-V5 construct was derived by PCR from the V5-p.1180_1504 construct and encodes both N- and C-terminal V5 epitope tags in the pcDNA3.1/V5-his mammalian expression vector (Invitrogen, Carlsbad, USA). To improve translation efficiency, the sequence surrounding the initiation codon was altered to correspond to the Kozak consensus.

We attempted to introduce nucleotide changes into an expression construct encoding full-length NF, kindly provided by Prof. Elgersma (Erasmus Medical Center, Rotterdam, The Netherlands). This was unsuccessful, most likely due to the large size and high GC content of the construct. Instead we derived 2 expression constructs encoding a truncated form of NF with a C-terminal myc-epitope tag: NF p.1_2069-myc and NF p.1_2069ins10-myc. First, a 6.2 kb fragment corresponding to *NF1* [NM_000267.3] c.1_6207 was obtained by HindIII digestion of the full-length NF construct and cloned into the pcDNA3.1 (-) myc mammalian expression vector (Invitrogen). The full-length construct encodes 10 amino acids corresponding to a brain-specific *NF1* exon, ch17: g.29530120_29530149 (GRCh37 (hg19), NM_000267.3 c.1260+1617_c.1260+1646, p.Asn420insSerThrPheLysHisGlyLeuGlyThrAla, that is not present in the processed NM_000267.3 transcript [38]. We referred to the resulting myc-tagged expression construct as NF p.1_2069ins10-myc. To obtain a wild-type construct encoding only amino acids 1 - 2069 of the *NF1* NM_000267.3 reference transcript, the sequence encoding the 10 amino acid insertion was removed by SDM to yield NF p.1_2069-myc.

Expression constructs encoding *NF1* variants were derived by SDM of the NF p.1180_1504-V5 [24], NF V5-p.1180_1504-V5, NF p.1_2069-myc or NF p.1_2069ins10-myc wild-type constructs. In each case, the entire open-reading frame of the construct was sequenced to confirm the presence of the desired change, and to ensure that no other non-synonymous changes were introduced by the SDM procedure. At least 2 independent, verified clones per variant were used to prepare separate plasmid DNA stocks. These duplicate stocks were subsequently tested individually in the transfection experiments. We did not experience problems with *NF1* expression plasmid stability or toxicity during prokaryote or eukaryote cell culture. In a few cases where the yield from a specific plasmid preparation was low (< 50 µg plasmid DNA from a 50 ml liquid culture), results were sub-optimal. In these cases, re-inoculation and isolation of a new preparation was sufficient to obtain high-quality DNA and reliable data.

The expression construct encoding full-length FLAG-tagged SPRED1 was kindly provided by Prof. Yoshimura (Keio University, Tokyo, Japan). The bacterial expression construct encoding the RAS-GTP binding domain (RBD) of RAF fused to glutathione-S-transferase (GST), GST-RAF-RBD, was kindly provided by Prof. Zwartkruis (Utrecht University, Utrecht, The Netherlands). Recombinant GST-RAF-RBD was prepared as described previously [35]. The HA-H-RAS expression construct [47] was purchased from Addgene (#39503; Cambridge, USA). The expression construct encoding GFP-TSC2 has been described previously [48].

Antibodies were purchased from Cell Signaling Technology (Danvers, USA)(rabbit anti-HA; mouse anti-HA, 9B11 mouse anti-myc), Invitrogen (mouse anti-V5) Sigma-Aldrich (St. Louis, USA) (mouse and rabbit anti-FLAG) and Li-Cor Biosciences (Lincoln, USA)( goat anti-rabbit 680 nm and goat anti-mouse 800 nm conjugates). Anti-FLAG affinity beads were purchased from Sigma-Aldrich, glutathione-sepharose was obtained from GE Healthcare (Uppsala, Sweden).

HEK 293T and COS-7 cells were maintained in Dulbecco's Modified Eagle Medium (DMEM)(Lonza, Verviers, Belgium) containing 10% fetal calf serum, 50 U/ml penicillin and 50 μg/ml streptomycin in a humidified 37^o^C, 5 - 10% CO_2_ incubator. The condition of the cells was critical for the success of the transfection experiments. Low passage number, exponentially growing subconfluent cells were essential to ensure a high transfection efficiency and obtain sufficient protein expression for detection and reliable immunoblot quantification.

*Assessment of the effects of NF1 variants on NF1 pre-mRNA splicing in patient material*

Total RNA was extracted from peripheral blood samples stored in PAX collection tubes using the Paxgene blood RNA kit (Qiagen, Venlo, The Netherlands) as recommended by the supplier. For isolation of total RNA from skin-derived fibroblast cultures, cells were cultured under standard conditions and in the presence of cycloheximide (overnight, 100 µg/ml) to inhibit nonsense-mediated mRNA decay. RNA was isolated using the RNeasy mini kit (Qiagen, Venlo, The Netherlands). Reverse transcriptase (RT) PCR was performed on 1 - 2 µg total RNA using the iScript cDNA synthesis kit (Bio-Rad), followed by PCR using primers specific for the variant to be analysed (primer sequences available on request). RT-PCR products were analysed by agarose gel electrophoresis and Sanger sequencing.

*In vitro assessment of the effects of NF1 variants on NF1 pre-mRNA splicing*

*In vitro* exon trap experiments were performed as described previously [33]. Briefly, 3 - 5 x 10^5^ HEK 293T cells were seeded per well of a 12- or 6-well culture dish and transfected the following day with 8 µg polyethyleneimine and 1.6 µg of the wild-type, variant or control (empty vector) pSPL3 splicing constructs. Transfection efficiency was monitored using a GFP-TSC2 expression construct: the proportion of GFP-TSC2 expressing cells was estimated by fluorescent microscopy. RNA was isolated 24 - 48 hours after transfection using the RNeasy mini kit (Qiagen, Venlo, The Netherlands). Reverse transcriptase (RT) PCR was performed using the iScript cDNA synthesis kit (Bio-Rad) and primers specific for pSPL3-derived transcripts: SD6_f 5'-ctgagtcacctggacaacc-3' and SA2_r 5'-atctcagtggtatttgtgagc-3'. PCR products were analysed by agarose gel electrophoresis and Sanger sequencing.

*In vitro assessment of RAS GAP activity*

To estimate RAS GAP activity, 2 - 3 x 10^5^ COS-7 or HEK 293T cells were seeded per well of a 6-well culture dish. The following day the cells were transfected with 2 µg of a 1:19 mixture of expression constructs encoding HA-H-RAS and wild-type or variant NF using Lipofectamine 2000 (Invitrogen). Transfection efficiency was monitored using a GFP-TSC2 expression construct, as above. In addition, GFP-TSC2 signals were estimated by immunoblotting. Twenty-four hours after transfection the growth medium was replaced with DMEM without additives for 4 hours. Subsequently the cells were stimulated with 10 ng/µl epidermal growth factor (EGF) (PeproTech, London, UK) for 5 minutes prior to lysis. Cells were transferred to ice, washed briefly with PBS (4^o^C) and lysed in 300 µl GAP lysis buffer (50 mM Tris-HCl (pH 7.6), 150 mM NaCl, 5 mM MgCl_2_, 1% (v/v) Triton X100, 1 mM DTT, 100 µM GDP and Complete^TM^ Ultra EDTA-free protease inhibitors (Roche Molecular Biochemicals, Woerden, The Netherlands)) for 5 minutes. After centrifugation (10 000 g, 10 minutes, 4^o^C), the cleared supernatant fractions were transferred to 15 µl of pre-washed and equilibrated glutathione-agarose beads coated with the recombinant GST-RAF-RBD. After gentle agitation for > 2 hours at 4^o^C, the beads were washed 3 times with > 20-fold excess of GAP lysis buffer and resuspended in sample buffer (62.5 mM Tris-HCl, pH 6.8, 2% sodium dodecyl sulphate (SDS), 10% glycerol, 2.5% β-mercaptoethanol, 0.01% bromophenol blue) prior to SDS-PAGE and immunoblot analysis using Criterion stain-free SDS-PAGE gels, nitrocellulose membranes and the Turboblotter semi-dry blotting apparatus (Bio-Rad, Hercules, USA). GST-RAF-RBD levels were estimated directly from the SDS-PAGE gel using the GelDoc gel imaging apparatus and quantification software (Bio-Rad). Immunoblots were visualised and signals corresponding to the specific protein bands were quantified using an Odyssey infra-red scanner and software (Li-Cor Biosciences).

RAS-GTP signals in the presence of the expressed variants were determined, relative to the signals in the absence of NF (control; = 1.0) and, to estimate the RAS GAP activity of the variants, these values were compared to the values for the wild-type NF protein. We applied the following formula:

relative RAS-GTP signal_wild-type_

RAS GAP activity_variant_ = ________________________

relative RAS-GTP signal_variant_

*In vitro assessment of the NF1-SPRED interaction*

To investigate the NF-SPRED interaction, 2 - 3 x 10^6^ HEK 293T cells were seeded into 10 cm diameter culture dishes. The following day the cells were transfected with 10 µg of a 1:9 mixture of the FLAG-SPRED1 and either wild-type or variant NF p.1_2069-myc or p.1_2069ins10-myc expression constructs. Transfection efficiency was monitored as above. Cells were harvested 48 hours after transfection in 800 µl lysis buffer (150 mM Tris-HCl pH 7.6, 50 mM NaCl, 1 mM EDTA, 0.5% Triton X100 and protease inhibitors (Complete^TM^, Roche Molecular Biochemicals). After centrifugation (10 000 g, 10 minutes, 4^o^C), the cleared supernatant fractions were transferred to 15 µl pre-washed and equilibrated anti-FLAG affinity beads (Sigma-Aldrich) and agitated gently for > 2 hours at 4^o^C. The beads were then washed 3 times with > 10-fold excess of wash buffer (50 mM Tris-HCl pH 7.6, 150 mM NaCl, 0.5% Triton X100) and resuspended in sample buffer prior to SDS-PAGE and immunoblot analysis. Immunoblots were analysed as above.

*References*

References 1 to 46, see main article.

1. P. Rodriguez-Viciana, P. H. Warne, A. Khwaja, B. M. Marte, D. Pappin, P. Das, M. D. Waterfield, A. Ridley, and J. Downward, “Role of phosphoinositide 3-OH kinase in cell transformation and control of the actin cytoskeleton by Ras,” Cell, vol. 89, no. 3, pp. 457–467, 1997.
2. M. Hoogeveen-Westerveld, L. van Unen, A. van den Ouweland, D. Halley, A. Hoogeveen, and M. Nellist, “The TSC1-TSC2 complex consists of multiple TSC1 and TSC2 subunits,” BMC Biochemistry, vol. 13, no. 1, article e18, 2012.
